# Efficacy and safety of esaxerenone vs trichlormethiazide for the treatment of uncontrolled essential hypertension in Japanese patients with type 2 diabetes mellitus: a subanalysis of the EXCITE-HT study

**DOI:** 10.1038/s41440-025-02437-z

**Published:** 2025-12-23

**Authors:** Mitsuru Ohishi, Kazuomi Kario, Tomohiro Katsuya, Tatsuo Shimosawa, Kazuhito Shiosakai, Taketoshi Furugori, Takashi Taguchi

**Affiliations:** 1 Department of Cardiovascular Medicine and Hypertension, Graduate School of Medical and Dental Sciences, Kagoshima University, Kagoshima, Japan; 2 Division of Cardiovascular Medicine, Department of Medicine, Jichi Medical University School of Medicine, Shimotsuke, Tochigi, Japan; 3 Katsuya Clinic, Amagasaki, Hyogo, Japan; 4 Department of Clinical Laboratory, School of Medicine, International University of Health and Welfare, Narita, Chiba, Japan; 5 Data Intelligence Department, Daiichi Sankyo Co., Ltd., Shinagawa-ku, Tokyo, Japan; 6 Primary Medical Science Department, Medical Affairs Division, Daiichi Sankyo Co., Ltd., Chuo-ku, Tokyo, Japan

**Keywords:** Esaxerenone, Japan, Morning home blood pressure, Trichlormethiazide, Type 2 diabetes mellitus

## Abstract

This subgroup analysis of the randomized, open-label, parallel-group EXCITE-HT study explored the antihypertensive efficacy and safety of esaxerenone vs trichlormethiazide in patients with type 2 diabetes mellitus (T2DM), stratified by baseline antihypertensive agent (angiotensin receptor blocker [ARB] or calcium channel blocker [CCB]) and urinary albumin-to-creatinine ratio (UACR; <30 or ≥30 mg/gCr). Using thresholds consistent with those used in the main study to interpret the difference in systolic/diastolic blood pressure (SBP/DBP), the between-group difference in least squares mean change (95% confidence interval [CI]) in morning home SBP/DBP at the end of treatment was −2.5 (−4.8, −0.2)/ − 0.7 (−2.0, 0.6) mmHg. Trends were consistent across all subgroups. The geometric mean UACR significantly decreased from baseline to Week 12 in the overall population, ARB subgroup (except for esaxerenone-treated patients), CCB subgroup, and both UACR subgroups. The overall incidence of serum potassium ≥5.5 mEq/L was 2.5% with esaxerenone and 0.9% with trichlormethiazide, with no cases of serum potassium ≥6.0 mEq/L. In this patient population, esaxerenone had a favorable safety profile, achieved blood pressure lowering similar to trichlormethiazide, and elicited a reduction of kidney damage (based on UACR), regardless of baseline antihypertensive agent or UACR.

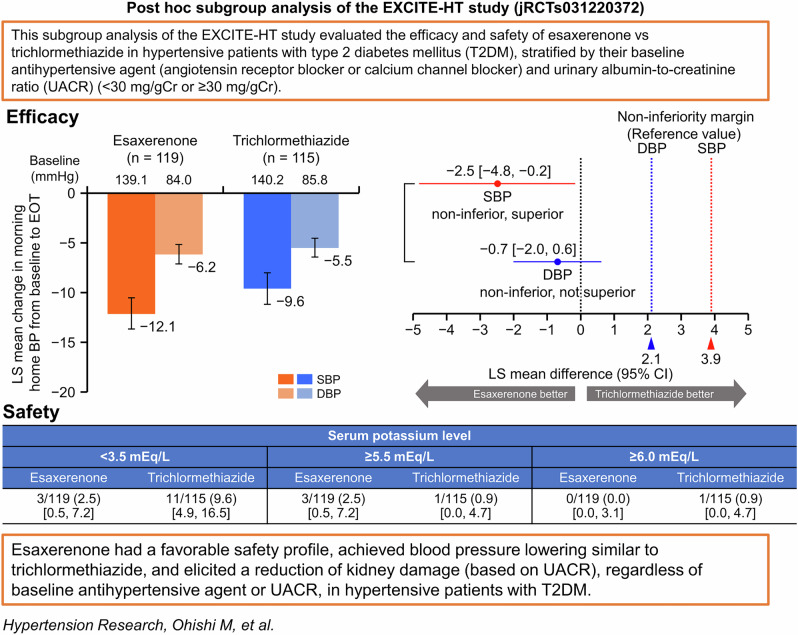

## Introduction

Hypertension is a major risk factor for cardiovascular and cerebrovascular morbidity and mortality and plays a critical role in the progression of diabetic nephropathy [1–9]. Patients often present with both hypertension and type 2 diabetes mellitus (T2DM), and their coexistence is projected to rise with an aging global population [10–12]. In patients with both hypertension and T2DM-related complications, stricter blood pressure (BP) control is beneficial because these patients are at higher risk of cardiovascular and kidney events [9, 13–15].

In Japan, the recommended first-line pharmacotherapy for hypertension includes calcium channel blockers (CCBs), angiotensin II receptor blockers (ARBs), angiotensin-converting enzyme inhibitors, and a low-dose diuretic (e.g., a thiazide or thiazide-like diuretic) [12]. For low-dose diuretics, regular assessment of kidney function (e.g., estimated glomerular filtration rate [eGFR] and urinary albumin) is recommended. Mineralocorticoid receptor blockers (MRBs) can be used in conjunction with angiotensin-converting enzyme inhibitors or ARBs for comprehensive management.

Esaxerenone is a novel, non-steroidal MRB with a higher selectivity for mineralocorticoid receptors, higher potency and bioavailability, and longer half-life than other MRBs [16, 17]. Clinical studies have demonstrated that esaxerenone (2.5 mg/day) is non-inferior to eplerenone (50 mg/day) in BP reduction [18], and has favorable renoprotective effects (reduction and remission of albuminuria) in hypertensive patients with T2DM [19–22]. These effects of esaxerenone in patients with T2DM are consistent regardless of concomitant use of sodium–glucose cotransporter-2 inhibitors [23, 24].

In the overall EXCITE-HT study, esaxerenone was non-inferior to trichlormethiazide in lowering morning home BP, with a relatively low incidence of hyperkalemia (serum potassium ≥5.5 mEq/L, 2.0%) compared with other esaxerenone studies [25–29]. However, data specifically on T2DM patients within the EXCITE-HT study have not yet been reported.

Therefore, this pre-specified subgroup analysis evaluated the efficacy and safety of esaxerenone vs trichlormethiazide in hypertensive patients with T2DM, stratified by their baseline antihypertensive agent (ARB or CCB) and urinary albumin-to-creatinine ratio (UACR) (<30 mg/gCr or ≥30 mg/gCr).

## Methods

### Study design

Details of the EXCITE-HT study have been published previously [25, 26]. Briefly, EXCITE-HT was a randomized, open-label, parallel-group study with 54 participating sites, conducted between December 2022 and September 2023. The present subgroup analysis of the EXCITE-HT study included hypertensive patients with co-existing T2DM.

The primary study protocol was approved by the Certified Review Board of Hattori Clinic (CRB3180027) and was registered in the Japan Registry of Clinical Trials (jRCTs031220372; https://jrct.mhlw.go.jp/en-latest-detail/jRCTs031220372). The study was conducted based on the principles of the Declaration of Helsinki and the Clinical Trials Act in Japan. The participants provided written informed consent before study enrollment.

### Patients

The full inclusion and exclusion criteria of the EXCITE-HT study have been reported previously [25]. Briefly, the EXCITE-HT study included patients aged ≥20 years who had received either one ARB or one CCB at the same dose for 4 or more weeks prior to registration and with mean morning home systolic BP (SBP) ≥ 125 mmHg and/or diastolic BP (DBP) ≥ 75 mmHg [25, 26]. Of the patients enrolled in the primary study, only those diagnosed with T2DM were included in this subanalysis.

### Study interventions

The starting dose of esaxerenone was 2.5 mg/day, except in patients with a creatinine-based eGFR (eGFR_creat_) 30–59 mL/min/1.73 m^2^ or those with T2DM and albuminuria at baseline, in whom the starting dose was reduced to 1.25 mg/day. Patients were administered esaxerenone for 12 weeks according to the Japanese package insert [30]. Based on BP and serum potassium level, the dose could be gradually increased to a maximum of 5 mg/day after 4 or 8 weeks of treatment, at the discretion of the attending physician.

Trichlormethiazide was administered at the discretion of the attending physician in line with the Japanese package insert and 2019 Japanese Society of Hypertension (JSH) guidelines [12, 31], which recommend a starting dose of ≤1 mg/day. The dose could be increased after 4 or 8 weeks of treatment at the physician’s discretion, based on the individual patient’s condition.

The dose of ARB or CCB (baseline antihypertensive agent) was maintained throughout the treatment period until the end of treatment (EOT), and the use of other antihypertensive agents was prohibited.

### Study endpoints

The primary endpoint was the change in morning home SBP/DBP from baseline to EOT in patients with T2DM. The secondary endpoints were the change in bedtime home and office SBP/DBP from baseline to EOT and the change in UACR and serum N-terminal pro-brain natriuretic peptide (NT-proBNP) levels from baseline to Week 12 in patients with T2DM.

The safety endpoints were as follows: treatment-emergent adverse events (TEAEs) coded using the Medical Dictionary for Regulatory Activities (MedDRA)/Japanese, version 25.1; time course changes and change from baseline in eGFR_creat_ and serum potassium level throughout the study period; proportion of patients with serum potassium level ≤3.5 mEq/L, ≥5.5 mEq/L, or ≥6.0 mEq/L; and proportion of patients with uric acid (UA) level >7.0 mg/dL.

### Sample size and statistical analyses

No sample size calculations were performed specifically for this subanalysis; the sample size was determined based on the primary study requirements. Data were analyzed post hoc by baseline antihypertensive agent (ARB or CCB) and baseline UACR (<30 mg/gCr [low UACR subgroup] or ≥30 mg/gCr [high UACR subgroup]).

As this subanalysis was exploratory, the same criteria used in the main analysis were used to evaluate the non-inferiority of esaxerenone to trichlormethiazide [25, 26]. In this subanalysis, the non-inferiority criteria established in the primary analysis were used to evaluate non-inferiority, and the non-inferiority margins from the primary analysis (3.9 mmHg for SBP and 2.1 mmHg for DBP) were referenced when interpreting the subanalysis results. If the upper limit of the two-sided 95% CI was <0 mmHg, esaxerenone was judged to be superior to trichlormethiazide.

The full analysis set (FAS) was used for the efficacy analysis, the safety analysis set was used for the safety analysis, and the definitions for the FAS and safety analysis set have been previously reported [25]. Unless otherwise specified, all statistical analyses were performed using a 5% two-sided significance threshold. SAS version 9.4 or later (SAS Institute Inc., Cary, NC, USA) was used for the statistical analyses.

## Results

In the EXCITE-HT study, 600 patients were eligible and randomly assigned to the esaxerenone and trichlormethiazide groups as follows: the FAS included 295 and 290 patients, respectively, and the safety analysis set included 302 and 298 patients, respectively [26]. The present subanalysis of patients with T2DM included 119 and 115 patients in the esaxerenone and trichlormethiazide groups, respectively.

Table 1 and Supplementary Table 1 show the baseline demographic and clinical characteristics of patients with T2DM in the overall population and in subgroups by baseline antihypertensive agent and UACR. In the overall population, in the esaxerenone and trichlormethiazide groups, respectively, the mean ± standard deviation age was 66.4 ± 10.7 and 66.0 ± 10.5 years, and the proportion of patients with body mass index ≥25 kg/m^2^ was 52.1% (62/119) and 61.7% (71/115); the proportion of patients with dyslipidemia, 68.1% (81/119) and 66.1% (76/115); the proportion of patients with UACR ≥ 30 mg/gCr, 42.0% (50/119) and 46.1% (53/115); and the proportion of patients with eGFR_creat_ 30 to <60 mL/min/1.73 m^2^, 24.4% (29/119) and 28.3% (32/113) (Table 1). In the overall population and in all subgroups, baseline characteristics were well balanced between the two treatment groups, with some exceptions.

**Table 1:** Baseline patient demographic and clinical characteristics in the overall population and in subgroups by baseline antihypertensive agent (full analysis set)

Characteristics	Overall		ARB		CCB	
	Esaxerenone *n* = 119	Trichlormethiazide *n* = 115	Esaxerenone *n* = 53	Trichlormethiazide *n* = 54	Esaxerenone *n* = 66	Trichlormethiazide *n* = 61
Sex, male	69 (58.0)	68 (59.1)	33 (62.3)	32 (59.3)	36 (54.5)	36 (59.0)
Age, years	66.4 ± 10.7	66.0 ± 10.5	66.1 ± 10.2	65.2 ± 9.5	66.8 ± 11.2	66.7 ± 11.3
≥65	69 (58.0)	66 (57.4)	27 (50.9)	29 (53.7)	42 (63.6)	37 (60.7)
Body mass index, kg/m^2^	26.27 ± 4.99	26.28 ± 4.07	26.37 ± 4.93	26.92 ± 4.15	26.19 ± 5.07	25.72 ± 3.95
≥25	62 (52.1)	71 (61.7)	29 (54.7)	36 (66.7)	33 (50.0)	35 (57.4)
Morning home SBP, mmHg	139.1 ± 14.4	140.2 ± 11.9	140.6 ± 15.4	141.9 ± 13.2	137.9 ± 13.5	138.7 ± 10.6
Morning home DBP, mmHg	84.0 ± 9.3	85.8 ± 9.2	84.9 ± 9.2	87.1 ± 9.6	83.3 ± 9.5	84.6 ± 8.6
Bedtime home SBP, mmHg	135.0 ± 15.1 *n* = 112	135.2 ± 13.6 *n* = 110	134.5 ± 17.2 *n* = 51	137.3 ± 15.8 *n* = 52	135.4 ± 13.3 *n* = 61	133.3 ± 11.1 *n* = 58
Bedtime home DBP, mmHg	79.7 ± 10.0 *n* = 112	80.0 ± 10.9 *n* = 110	79.7 ± 9.6 *n* = 51	81.0 ± 11.1 *n* = 52	79.6 ± 10.4 *n* = 61	79.1 ± 10.7 *n* = 58
Office SBP, mmHg	141.8 ± 14.9	141.7 ± 15.8	144.0 ± 17.3	145.3 ± 18.1	140.0 ± 12.5	138.5 ± 12.9
Office DBP, mmHg	80.8 ± 11.6	81.1 ± 10.6	80.6 ± 11.1	83.0 ± 9.5	80.9 ± 12.1	79.3 ± 11.3
NT-proBNP, pg/mL	77.95 ± 99.47 *n* = 98	87.47 ± 187.78 *n* = 95	93.28 ± 129.79 *n* = 43	110.64 ± 270.91 *n* = 42	65.96 ± 65.85 *n* = 55	69.11 ± 71.42 n = 53
<55	55 (56.1)	55 (57.9)	24 (55.8)	25 (59.5)	31 (56.4)	30 (56.6)
55 to <125	22 (22.4)	24 (25.3)	8 (18.6)	8 (19.0)	14 (25.5)	16 (30.2)
≥125	21 (21.4)	16 (16.8)	11 (25.6)	9 (21.4)	10 (18.2)	7 (13.2)
UACR, mg/gCr	165.96 ± 442.12	156.56 ± 637.29	192.41 ± 540.65	230.78 ± 916.54	144.72 ± 346.38	90.86 ± 142.36
<30	69 (58.0)	62 (53.9)	33 (62.3)	32 (59.3)	36 (54.5)	30 (49.2)
30 to <300	36 (30.3)	42 (36.5)	15 (28.3)	17 (31.5)	21 (31.8)	25 (41.0)
≥300	14 (11.8)	11 (9.6)	5 (9.4)	5 (9.3)	9 (13.6)	6 (9.8)
Serum potassium, mEq/L	4.26 ± 0.33 *n* = 115	4.26 ± 0.33 *n* = 110	4.35 ± 0.32 *n* = 51	4.32 ± 0.32 *n* = 51	4.19 ± 0.33 *n* = 64	4.21 ± 0.32 *n* = 59
Uric acid, mg/dL	5.23 ± 1.20 *n* = 119	5.12 ± 1.21 *n* = 114	5.25 ± 1.18 *n* = 53	5.28 ± 1.18 *n* = 54	5.22 ± 1.22 *n* = 66	4.98 ± 1.22 *n* = 60
eGFR_creat_, mL/min/1.73 m^2^	72.81 ± 17.50 *n* = 119	70.79 ± 17.37 *n* = 113	74.87 ± 18.07 *n* = 53	67.66 ± 19.03 *n* = 54	71.16 ± 16.99 *n* = 66	73.66 ± 15.30 *n* = 59
30 to <60	29 (24.4)	32 (28.3)	12 (22.6)	20 (37.0)	17 (25.8)	12 (20.3)
≥60	90 (75.6)	81 (71.7)	41 (77.4)	34 (63.0)	49 (74.2)	47 (79.7)
Duration of hypertension, years	6.94 ± 5.73 *n* = 70	5.90 ± 5.08 *n* = 63	6.53 ± 5.31 *n* = 34	6.59 ± 5.18 *n* = 29	7.33 ± 6.15 *n* = 36	5.32 ± 5.00 n = 34
Complication						
Dyslipidemia	81 (68.1)	76 (66.1)	31 (58.5)	44 (81.5)	50 (75.8)	32 (52.5)
Hyperuricemia	18 (15.1)	10 (8.7)	7 (13.2)	7 (13.0)	11 (16.7)	3 (4.9)
Heart failure	15 (12.6)	10 (8.7)	9 (17.0)	4 (7.4)	6 (9.1)	6 (9.8)
Esaxerenone dose at baseline (initial dose), mg						
1.25	69 (58.0)		28 (52.8)		41 (62.1)	
2.5	50 (42.0)		25 (47.2)		25 (37.9)	
Esaxerenone dose at EOT (last dose), mg						
1.25	36 (30.3)		16 (30.2)		20 (30.3)	
2.5	68 (57.1)		32 (60.4)		36 (54.5)	
5	15 (12.6)		5 (9.4)		10 (15.2)	
Trichlormethiazide dose at baseline (initial dose), mg						
0.25		3 (2.6)		1 (1.9)		2 (3.3)
0.5		6 (5.2)		3 (5.6)		3 (4.9)
1		106 (92.2)		50 (92.6)		56 (91.8)
2		0		0		0
Trichlormethiazide dose at EOT (last dose), mg						
0.25		1 (0.9)		0		1 (1.6)
0.5		7 (6.1)		4 (7.4)		3 (4.9)
1		96 (83.5)		46 (85.2)		50 (82.0)
>1 to ≤2		11 (9.6)		4 (7.4)		7 (11.5)
≥3		0		0		0

Data are n (%) or mean ± standard deviation

Data for the ARB and CCB subgroups are from a post hoc analysis

*ARB* angiotensin II receptor blocker, *CCB* calcium channel blocker, *DBP* diastolic blood pressure, *eGFR*_*creat*_ creatinine-based estimated glomerular filtration rate, *EOT* end of treatment, *NT-proBNP* N-terminal pro-brain natriuretic peptide, *SBP* systolic blood pressure, *UACR* urinary albumin-to-creatinine ratio

### BP-lowering effects

**Fig. 1:**
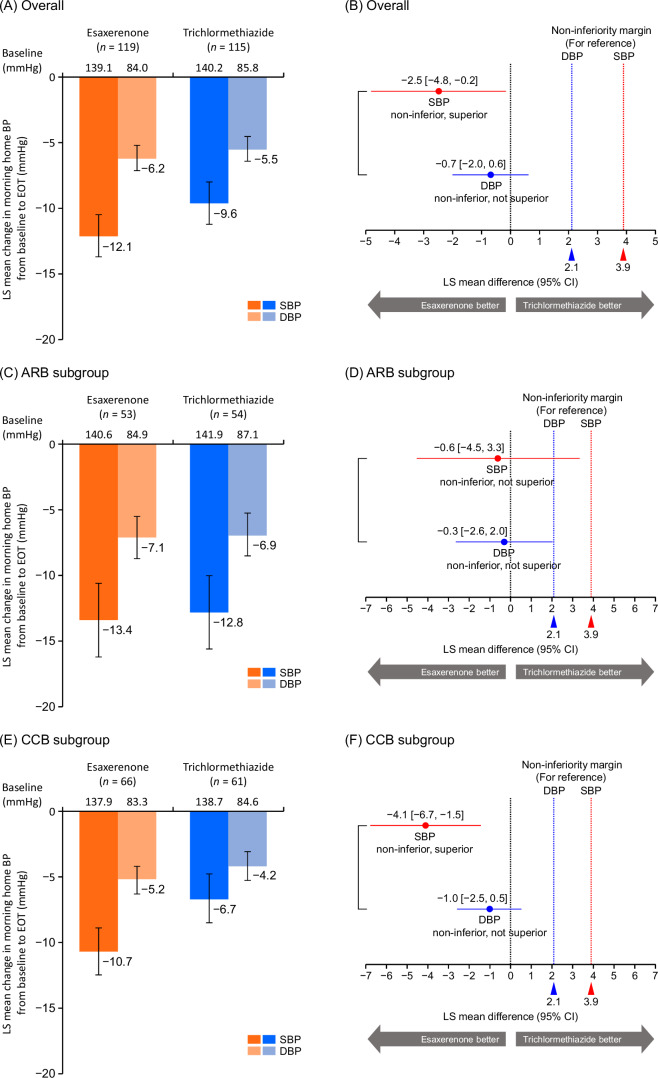

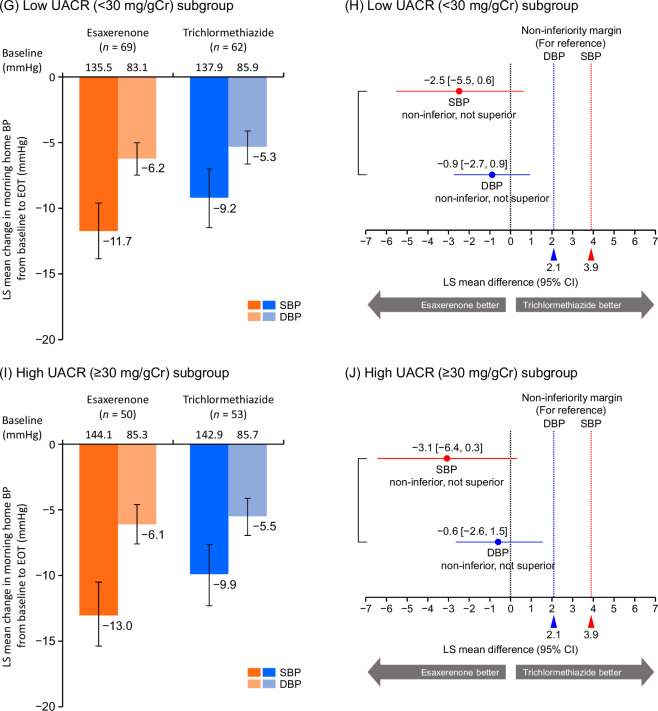
Changes from baseline to EOT in morning home BP (full analysis set). **A, B** overall population, **C, D** ARB subgroup, **E, F** CCB subgroup, **G, H** low UACR (<30 mg/gGr) subgroup, and **I, J** high UACR (≥30 mg/gCr) subgroup. For panels **B, D, F,** **H**, and **J**, the red dotted line (3.9 mmHg) and blue dotted line (2.1 mmHg) indicate the non-inferiority criteria. Data are LS mean (95% CI). Data for the ARB, CCB, and baseline UACR subgroups are from a post hoc analysis. *ARB* angiotensin II receptor blocker, *BP* blood pressure, *CCB* calcium channel blocker, *CI* confidence interval, *DBP* diastolic blood pressure, *EOT* end of treatment, *LS* least squares, *SBP* systolic blood pressure, *UACR* urinary albumin-to-creatinine ratio

Morning home SBP/DBP significantly decreased from baseline to EOT in all subgroups (Supplementary Tables 2 and 3). In the overall population, the least squares (LS) mean changes in morning home SBP/DBP from baseline to EOT were −12.1 (95% CI, −13.7, −10.5)/ − 6.2 (−7.1, −5.2) mmHg in the esaxerenone group and −9.6 (−11.2, −8.0)/ −5.5 (−6.4, −4.5) mmHg in the trichlormethiazide group (Fig. 1A). The differences in LS mean change in morning home SBP/DBP between the two groups at EOT were −2.5 (−4.8, −0.2)/ −0.7 (−2.0, 0.6) mmHg (Fig. 1B), confirming the non-inferiority of esaxerenone to trichlormethiazide in reducing both morning home SBP and DBP, and superiority in reducing morning home SBP but not DBP. The changes from baseline to EOT in morning home BP in the population without T2DM are shown in Supplementary Fig. 1.

Similar trends were observed in both ARB and CCB subgroups and both UACR subgroups (Fig. 1C–J). In the CCB subgroup, esaxerenone was superior to trichlormethiazide in reducing morning home SBP but not DBP, whereas the ARB subgroup did not show this trend for superiority. Significant reductions were also shown in bedtime home and office SBP/DBP in both treatment groups in the overall population and all subgroups (all *P* < 0.01) (Supplementary Tables 2 and 3). The changes from baseline in morning home BP, bedtime home BP, and office BP in the population without T2DM are summarized in Supplementary Table 4.

### UACR

**Fig. 2:**
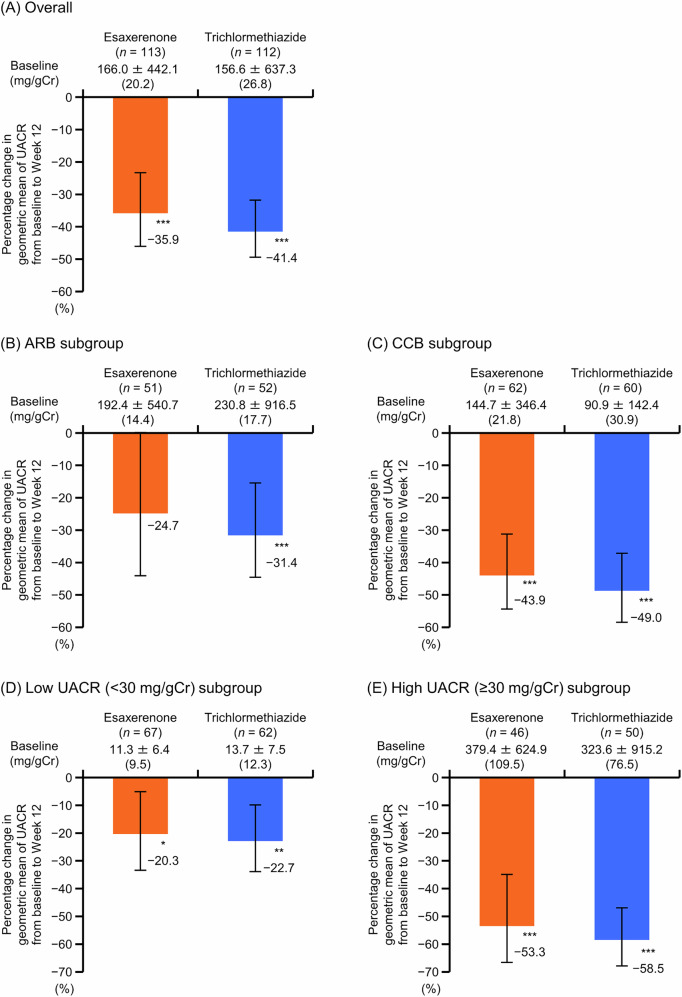
Percentage change in geometric mean of UACR from baseline to Week 12 (full analysis set). **A** overall population, **B** ARB subgroup, **C** CCB subgroup, **D** low UACR (<30 mg/gGr) subgroup, and **E** high UACR (≥30 mg/gCr) subgroup. Data are shown as geometric mean ± standard deviation (median) for baseline UACR (above the graphs) and percentage change in UACR with 95% CIs (bar graphs). *P < 0.05 versus baseline, paired *t*-test. **P < 0.01 versus baseline, paired *t*-test. ***P < 0.001 versus baseline, paired *t*-test. Data for the ARB, CCB, and baseline UACR subgroups are from a post hoc analysis. *ARB* angiotensin II receptor blocker, *CCB* calcium channel blocker, *CI* confidence interval, *UACR* urinary albumin-to-creatinine ratio

The geometric mean of UACR significantly decreased from baseline to Week 12 in the overall population (−35.9% for esaxerenone, −41.4% for trichlormethiazide; both *P* < 0.001 vs baseline); the ARB and CCB subgroups, except for the ARB subgroup treated with esaxerenone (ARB subgroup: −24.7% for esaxerenone [*P* = 0.066] and −31.4% for trichlormethiazide; CCB subgroup, −43.9% for esaxerenone and −49.0% for trichlormethiazide; all *P* < 0.001 vs baseline except the ARB subgroup treated with esaxerenone); and the UACR subgroups (low UACR subgroup: −20.3% for esaxerenone [*P* < 0.05 vs baseline] and −22.7% for trichlormethiazide [*P* < 0.01 vs baseline]; high UACR subgroup: −53.3% for esaxerenone and −58.5% for trichlormethiazide [both *P* < 0.001 vs baseline]) (Fig. 2 and Supplementary Tables 5 and 6). The changes from baseline in the geometric mean of UACR were numerically greater in the CCB subgroup compared with the ARB subgroup, regardless of whether patients received esaxerenone or trichlormethiazide. The change in UACR from baseline to Week 12 in the population without T2DM is shown in Supplementary Table 7.

### Safety

TEAEs in the overall population and in subgroups by baseline antihypertensive agent and UACR are shown in Supplementary Tables 8 and 9. In the overall population, TEAEs occurred in 40 (32.8%) and 33 (27.5%) patients in the esaxerenone and trichlormethiazide groups, respectively. Among the overall population, blood potassium increased and blood potassium decreased in four (3.3%) and 0 patients treated with esaxerenone and in 0 and one (0.8%) patient treated with trichlormethiazide, respectively. No cases of hyperkalemia or hypokalemia were reported in any group. Hyperuricemia was reported in one (0.8%) and four (3.3%) patients treated with esaxerenone and trichlormethiazide, respectively.

### eGFR_creat_

Time course changes in eGFR_creat_ and serum potassium levels in the overall population and subgroups by baseline antihypertensive agent and UACR are shown in Supplementary Fig. 2 and Supplementary Tables 10 and 11. eGFR_creat_ decreased over the first 2 weeks and remained relatively stable until Week 12 in the overall population and all subgroups by baseline antihypertensive agent and UACR (Supplementary Fig. 2). No clinically meaningful differences were observed between subgroups or treatments. The changes in eGFR_creat_ from baseline to Week 12 were −6.88 ± 9.94 and −3.00 ± 9.25 mL/min/1.73 m^2^ in the esaxerenone and trichlormethiazide groups, respectively, within the ARB subgroup; and −6.16 ± 8.42 and −2.77 ± 9.65 mL/min/1.73 m^2^, respectively, within the CCB subgroup (Supplementary Table 10). The changes in eGFR_creat_ from baseline to Week 12 were −6.76 ± 9.72 and −1.87 ± 10.00 mL/min/1.73 m^2^ in the esaxerenone and trichlormethiazide groups, respectively, within the low UACR subgroup; and −6.09 ± 8.22 and −4.18 ± 8.54 mL/min/1.73 m^2^, respectively, within the high UACR subgroup (Supplementary Table 11).

### Serum potassium

In the esaxerenone group, serum potassium levels increased over the first 2 weeks then remained generally constant until Week 12 after starting esaxerenone treatment in the overall population and all subgroups by baseline antihypertensive agent and UACR (Supplementary Fig. 3). In the trichlormethiazide group, serum potassium levels gradually decreased regardless of baseline antihypertensive treatment and UACR. Among the overall population, the changes in serum potassium from baseline to Week 12 were 0.05 ± 0.36 and −0.21 ± 0.38 mEq/L in the esaxerenone and trichlormethiazide groups, respectively (Supplementary Table 10).

The incidence of serum potassium level <3.5 mEq/L was lower in the esaxerenone group compared with the trichlormethiazide group in the overall population (2.5% and 9.6%, respectively) (Table 2). This trend was pronounced in the CCB subgroup (1.5% and 14.3%, respectively) and high UACR subgroup (0.0% and 14.8%, respectively). The incidences of serum potassium level <3.5 mEq/L were comparable between the esaxerenone and trichlormethiazide groups within the ARB and low UACR subgroups. The incidence of serum potassium level ≥5.5 mEq/L was higher in the esaxerenone group compared with the trichlormethiazide group in the overall population (2.5% and 0.9%, respectively) and all subgroups except for the CCB subgroup (1.5% and 1.6%, respectively). No cases of serum potassium level ≥6.0 mEq/L were observed in any of the subgroups by baseline antihypertensive agent or UACR.

**Table 2:** Incidence of serum potassium level <3.5, ≥5.5, and ≥6.0 mEq/L (safety analysis set)

	Serum potassium level					
	<3.5 mEq/L		≥5.5 mEq/L		≥6.0 mEq/L	
	Esaxerenone	Trichlormethiazide	Esaxerenone	Trichlormethiazide	Esaxerenone	Trichlormethiazide
**Overall**	3/119 (2.5) [0.5, 7.2]	11/115 (9.6) [4.9, 16.5]	3/119 (2.5) [0.5, 7.2]	1/115 (0.9) [0.0, 4.7]	0/119 (0.0) [0.0, 3.1]	0/115 (0.0) [0.0, 3.2]
**ARB**	2/54 (3.7) [0.5, 12.7]	2/52 (3.8) [0.5, 13.2]	2/54 (3.7) [0.5, 12.7]	0/52 (0.0) [0.0, 6.8]	0/54 (0.0) [0.0, 6.6]	0/52 (0.0) [0.0, 6.8]
**CCB**	1/65 (1.5) [0.0, 8.3]	9/63 (14.3) [6.7, 25.4]	1/65 (1.5) [0.0, 8.3]	1/63 (1.6) [0.0, 8.5]	0/65 (0.0) [0.0, 5.5]	0/63 (0.0) [0.0, 5.7]
**UACR** < **30 mg/gCr**	3/70 (4.3) [0.9, 12.0]	3/61 (4.9) [1.0, 13.7]	2/70 (2.9) [0.3, 9.9]	1/61 (1.6) [0.0, 8.8]	0/70 (0.0) [0.0, 5.1]	0/61 (0.0) [0.0, 5.9]
**UACR** ≥ **30 mg/gCr**	0/49 (0.0) [0.0, 7.3]	8/54 (14.8) [6.6, 27.1]	1/49 (2.0) [0.1, 10.9]	0/54 (0.0) [0.0, 6.6]	0/49 (0.0) [0.0, 7.3]	0/54 (0.0) [0.0, 6.6]

Data are n/N (%) [95% CI]

Data for the ARB, CCB, and baseline UACR subgroups are from a post hoc analysis

*ARB* angiotensin II receptor blocker, *CCB* calcium channel blocker, *CI* confidence interval, *UACR* urinary albumin-to-creatinine ratio

### UA levels

The incidence of UA level >7.0 mg/dL was higher in the trichlormethiazide group compared with the esaxerenone group in the overall population (26.7% and 20.5%, respectively) (Table 3). This tendency was more notable in the ARB subgroup (32.7% and 18.2%, respectively) and high UACR subgroup (30.4% and 21.6%, respectively). The incidence of UA level >7.0 mg/dL was similar between the trichlormethiazide and esaxerenone groups within the CCB subgroup (21.5% and 22.4%, respectively).

**Table 3:** Incidence of UA level >7.0 mg/dL (safety analysis set)

	Esaxerenone	Trichlormethiazide
**Overall**	25/122 (20.5) [13.7, 28.7]	32/120 (26.7) [19.0, 35.5]
**ARB**	10/55 (18.2) [9.1, 30.9]	18/55 (32.7) [20.7, 46.7]
**CCB**	15/67 (22.4) [13.1, 34.2]	14/65 (21.5) [12.3, 33.5]
**UACR** < **30 mg/gCr**	14/71 (19.7) [11.2, 30.9]	15/64 (23.4) [13.8, 35.7]
**UACR** ≥ **30 mg/gCr**	11/51 (21.6) [11.3, 35.3]	17/56 (30.4) [18.8, 44.1]

Data are n/N (%) [95% CI]

Data for the ARB, CCB, and baseline UACR subgroups are from a post hoc analysis

*ARB* angiotensin II receptor blocker, *CCB* calcium channel blocker, *CI* confidence interval, *UA* uric acid, *UACR* urinary albumin-to-creatinine ratio

## Discussion

In the present subanalysis of the EXCITE-HT study focusing on patients with T2DM, esaxerenone administered as a second-line antihypertensive agent showed BP-lowering effects that were generally comparable to those of trichlormethiazide, with a trend toward a greater reduction in morning SBP compared with DBP. To our knowledge, this is among the first reports to directly compare esaxerenone with trichlormethiazide specifically in patients with T2DM. These findings have important clinical implications for the management of patients with both hypertension and T2DM, particularly given the high risks of cardiovascular, kidney, and metabolic complications in this population.

The BP reductions seen in both the overall analysis and subgroups—classified by baseline antihypertensive agent (ARB or CCB) and baseline UACR levels (<30 or ≥30 mg/gCr)—were consistent, suggesting that esaxerenone elicits a reliable antihypertensive effect regardless of background therapy or baseline kidney function. This is especially relevant in clinical practice, where patients with T2DM may have varying degrees of albuminuria and may already be taking different classes of antihypertensive medications. Achieving strict BP targets can be challenging in patients with T2DM, and our results imply that esaxerenone may serve as a valuable second-line agent capable of reducing BP to recommended levels in difficult-to-control scenarios.

Although esaxerenone and trichlormethiazide achieve BP reduction via distinct natriuretic pathways (MR antagonism in the collecting duct vs sodium–chloride cotransporter inhibition in the distal tubule) their comparable antihypertensive efficacy observed in this study may reflect the importance of volume reduction in salt-sensitive populations such as Japanese patients with T2DM. Both agents increased plasma renin activity and aldosterone levels as compensatory responses to sodium loss; however, only esaxerenone inhibits MR signaling. These findings suggest that MR-mediated mechanisms may not be the predominant driver of baseline hypertension in some patients, while MR blockade may still contribute to organ protection through mechanisms beyond BP reduction.

Interestingly, our subgroup analyses suggest that combining diuretics—whether from the thiazide or MRB class—with ARBs may produce more substantial BP-lowering benefits than combinations with CCBs. This result is consistent with previous findings [27], potentially owing to the synergy of targeting the renin–angiotensin–aldosterone system and the sodium/water reabsorption pathways. This synergy may be particularly relevant in populations with a propensity for salt sensitivity, such as some patients with T2DM. While the antihypertensive effect of ARBs is attenuated by excessive salt intake and may be inadequate [32], diuretics inhibit sodium–chloride cotransporters in the distal tubule, reducing sodium reabsorption and improving salt sensitivity. The combination of diuretics and ARBs is recommended in the 2019 JSH guidelines [12]. However, our study showed that esaxerenone, unlike thiazide-type diuretics, does not induce hypokalemia or elevate UA levels, both of which can impose further cardiovascular risk over time. Thus, selecting an MRB over a thiazide diuretic might facilitate effective BP control while simultaneously mitigating metabolic disturbances. Such benefits may be especially important when balancing the risks of cardiovascular and kidney events in patients with T2DM, who often present with multiple comorbidities.

Our results also highlight the potential renoprotective effects of esaxerenone, as evidenced by its effect of reducing urinary albumin excretion. While both esaxerenone and trichlormethiazide reduced UACR, the esaxerenone group presented consistent benefits in reducing albuminuria, particularly in patients with a higher UACR at baseline. Although the sample size was modest and the treatment period was relatively short, which may preclude definitive conclusions about long-term kidney outcomes, these findings align with previous studies of esaxerenone in patients with albuminuria [19–22]. Considering that elevated UACR is an early indicator of diabetic nephropathy and a predictor of adverse cardiovascular events [33, 34], the albuminuria-lowering effects of esaxerenone represent an important advantage. Additional research with longer treatment duration or larger-scale prospective studies will help determine whether these acute improvements in albuminuria translate to sustained renoprotection in the long term.

Of particular note in terms of safety is that the development of hyperkalemia—a recognized risk during treatment with MRBs—was effectively minimized. Although diabetes is a recognized risk factor for hyperkalemia [29], no cases of serum potassium level ≥6.0 mEq/L occurred, and only three patients overall (2.5%) had mild to moderate elevations (≥5.5 mEq/L). This low occurrence of events related to serum potassium increased may be due to dose adjustments and regular potassium monitoring, as recommended in the package insert [29, 30]. This favorable finding might reflect both cautious selection of initial esaxerenone doses and close laboratory follow-up, which are critical steps when prescribing MRBs in T2DM. Future studies investigating long-term potassium management strategies, including dietary counseling and possible concomitant use of potassium-binding agents, would help define a comprehensive framework for esaxerenone use.

Conversely, although trichlormethiazide lowered BP effectively, its use was linked to frequent electrolyte imbalances, specifically hypokalemia, as well as increased UA levels. Furthermore, in the present analysis, the elevation of serum UA was attenuated in the esaxerenone group, resulting in a significantly lower incidence of hyperuricemia compared with the trichlormethiazide group. Esaxerenone is an MRB, whereas thiazide diuretics are more likely to increase UA levels by reducing UA excretion in the kidneys. Because esaxerenone does not share this mechanism, it may lead to a lower incidence of hyperuricemia. In patients with coexisting diabetes, considerations beyond kidney protection—particularly those related to metabolic health—are critical. Thus, the lower likelihood of esaxerenone to increase UA levels compared with diuretics may have significance for long-term risk management. The combination of hypokalemia and hyperuricemia may lead to a higher risk of clinical complications in certain individuals [35, 36], emphasizing the value of therapies that maintain serum potassium levels and do not promote hyperuricemia. Esaxerenone’s potential advantage over trichlormethiazide in maintaining potassium homeostasis suggests its favorable profile for longer-term risk management, although ongoing vigilance for hyperkalemia remains necessary.

The study limitations of this subgroup analysis include its exploratory nature and the relatively short follow-up period of 12 weeks, which may not fully capture long-term outcomes or potential side effects. Furthermore, these limitations were based on those identified in the primary study [25, 26], which may have limited their applicability to this subgroup analysis given the small number of cases. The baseline antihypertensive agents were prescribed at the discretion of the physicians under real-life clinical conditions, which may have introduced bias. Some patients with primary aldosteronism may have been included because a confirmed diagnosis of primary aldosteronism was not mandatory.

## Conclusion

In conclusion, these findings suggest that esaxerenone is an effective and generally well-tolerated treatment option for hypertensive patients with T2DM, offering a favorable profile for BP management and UACR reduction. By minimizing the incidence of hypokalemia and potentially providing renoprotective effects, esaxerenone shows promise in addressing the multifaceted challenges faced by patients with T2DM. Long-term cardiovascular outcomes are expected to be verified in larger-scale trials.

## Supplementary information

Supplementary information

## Data Availability

The anonymized data underlying the results presented in this manuscript may be made available to researchers upon submission of a reasonable request to the corresponding author. The decision to disclose the data will be made by the corresponding author and the funder, Daiichi Sankyo Co., Ltd. Data disclosure can be requested for 36 months from article publication.
