# Supplementary material for: Efficacy and safety of esaxerenone vs trichlormethiazide for the treatment of uncontrolled essential hypertension in Japanese patients with type 2 diabetes mellitus: a subanalysis of the EXCITE-HT study
Source: Hypertens Res. 2025 Dec 23;49(2):444–56. doi: 10.1038/s41440-025-02437-z (PMC12823434; doi:10.1038/s41440-025-02437-z)
Supplement: Supplementary file 1 — Supplementary information [file 41440_2025_2437_MOESM1_ESM.pdf]

Supplementary Table 1. Baseline patient demographic and clinical characteristics by baseline UACR (full analysis set)

	UACR <30 mg/gCr		UACR ≥30 mg/gCr	
**Characteristics**	**Esaxerenone**<br>**n = 69**	**Trichlormethiazide**<br>**n = 62**	**Esaxerenone**<br>**n = 50**	**Trichlormethiazide**<br>**n = 53**
Sex, male	43 (62.3)	36 (58.1)	26 (52.0)	32 (60.4)
Age, years	64.8 ± 10.7	65.0 ± 11.2	68.7 ± 10.4	67.1 ± 9.5
≥65	37 (53.6)	35 (56.5)	32 (64.0)	31 (58.5)
Body mass index, kg/m^2	26.92 ± 5.27	26.60 ± 3.76	25.37 ± 4.46	25.91 ± 4.42
≥25	37 (53.6)	42 (67.7)	25 (50.0)	29 (54.7)
Morning home SBP, mmHg	135.5 ± 11.8	137.9 ± 10.8	144.1 ± 16.2	142.9 ± 12.7
Morning home DBP, mmHg	83.1 ± 7.7	85.9 ± 8.0	85.3 ± 11.1	85.7 ± 10.4
Bedtime home SBP, mmHg	132.4 ± 13.3<br>n = 69	132.3 ± 11.0<br>n = 60	139.0 ± 17.0<br>n = 43	138.7 ± 15.6<br>n = 50
Bedtime home DBP, mmHg	78.4 ± 9.1<br>n = 69	79.6 ± 10.5<br>n = 60	81.7 ± 11.0<br>n = 43	80.5 ± 11.4<br>n = 50
Office SBP, mmHg	140.6 ± 14.0	136.2 ± 11.9	143.5 ± 15.9	148.1 ± 17.5
Office DBP, mmHg	81.1 ± 11.7	80.7 ± 10.2	80.3 ± 11.6	81.5 ± 11.2
NT-proBNP, pg/mL	54.29 ± 57.40<br>n = 54	62.72 ± 65.30<br>n = 51	106.98 ± 129.20<br>n = 44	116.17 ± 265.63<br>n = 44
<55	35 (64.8)	31 (60.8)	20 (45.5)	24 (54.5)
55 to <125	12 (22.2)	14 (27.5)	10 (22.7)	10 (22.7)
≥125	7 (13.0)	6 (11.8)	14 (31.8)	10 (22.7)
UACR, mg/gCr	11.31 ± 6.43	13.74 ± 7.47	379.37 ± 624.91	323.63 ± 915.17
Serum potassium, mEq/L	4.28 ± 0.33<br>n = 68	4.30 ± 0.33<br>n = 59	4.24 ± 0.34<br>n = 47	4.21 ± 0.32<br>n = 51
Uric acid, mg/dL	5.18 ± 1.08<br>n = 69	4.99 ± 1.08<br>n = 61	5.30 ± 1.35<br>n = 50	5.28 ± 1.33<br>n = 53
$eGFR_{creat}$, mL/min/1.73 m^2	74.40 ± 17.75<br>n = 69	73.88 ± 17.00<br>n = 62	70.61 ± 17.09<br>n = 50	67.04 ± 17.23<br>n = 51
30 to <60	13 (18.8)	15 (24.2)	16 (32.0)	17 (33.3)
≥60	56 (81.2)	47 (75.8)	34 (68.0)	34 (66.7)
Duration of hypertension,	5.71 ± 5.38	6.19 ± 5.12	8.40 ± 5.87	5.59 ± 5.11

years	n = 38	n = 33	n = 32	n = 30
Complication				
Dyslipidemia	45 (65.2)	42 (67.7)	36 (72.0)	34 (64.2)
Hyperuricemia	7 (10.1)	4 (6.5)	11 (22.0)	6 (11.3)
Heart failure	8 (11.6)	3 (4.8)	7 (14.0)	7 (13.2)
Esaxerenone dose at baseline (initial dose), mg				
1.25	21 (30.4)	-	48 (96.0)	-
2.5	48 (69.6)	-	2 (4.0)	-
Esaxerenone dose at EOT (last dose), mg				
1.25	18 (26.1)	-	18 (36.0)	-
2.5	44 (63.8)	-	24 (48.0)	-
5	7 (10.1)	-	8 (16.0)	-
Trichlormethiazide dose at baseline (initial dose), mg				
0.25	-	1 (1.6)	-	2 (3.8)
0.5	-	3 (4.8)	-	3 (5.7)
1	-	58 (93.5)	-	48 (90.6)
2	-	0	-	0
Trichlormethiazide dose at EOT (last dose), mg				
0.25	-	0	-	1 (1.9)
0.5	-	4 (6.5)	-	3 (5.7)
1	-	55 (88.7)	-	41 (77.4)
>1 to ≤2	-	3 (4.8)	-	8 (15.1)
≥3	-	0	-	0
Basal antihypertensive agents				
ARB	33 (47.8)	32 (51.6)	20 (40.0)	22 (41.5)
CCB	36 (52.2)	30 (48.4)	30 (60.0)	31 (58.5)

Data are n (%) or mean ± standard deviation.

Data are from a post hoc analysis.

ARB angiotensin II receptor blocker, *CCB* calcium channel blocker, *DBP* diastolic blood pressure, *eGFR*$_{creat}$ creatinine-based estimated glomerular filtration rate, *EOT* end of treatment, *NT-proBNP* N-terminal pro-brain natriuretic peptide, *SBP* systolic blood pressure, *T2DM* type 2 diabetes mellitus, *UACR* urinary albumin-to-creatinine ratio.

Supplementary Table 2. Change from baseline in BP in the overall population and in subgroups by baseline antihypertensive agent (full analysis set)

	Overall						ARB						CCB					
	Esaxerenone			Trichlormethiazide			Esaxerenone			Trichlormethiazide			Esaxerenone			Trichlormethiazide		
	n	SBP, mmHg	DBP, mmHg	n	SBP, mmHg	DBP, mmHg	n	SBP, mmHg	DBP, mmHg	n	SBP, mmHg	DBP, mmHg	n	SBP, mmHg	DBP, mmHg	n	SBP, mmHg	DBP, mmHg
Morning home BP																		
Baseline	119	139.1 ± 14.4	84.0 ± 9.3	115	140.2 ± 11.9	85.8 ± 9.2	53	140.6 ±15.4	84.9 ± 9.2	54	141.9 ± 13.2	87.1 ± 9.6	66	137.9 ± 13.5	83.3 ± 9.5	61	138.7 ± 10.6	84.6 ± 8.6
Week 12	112	127.2 ± 11.9	78.1 ± 8.9	113	130.5 ± 11.2	80.1 ± 8.6	51	128.1 ± 13.7	78.3 ± 8.9	52	128.9 ± 12.2	80.0 ± 9.5	61	126.4 ± 10.1	77.9 ± 9.0	61	131.9 ± 10.3	80.2 ± 7.8
Change from baseline	112	−12.1 ± 10.5	−6.1 ± 5.7	113	−9.4 ± 10.0	−5.5 ± 5.9	51	−13.2 ± 11.7	−6.8 ± 6.4	52	−12.4 ± 12.2	−6.9 ± 7.4	61	−11.1 ± 9.3	−5.5 ± 5.0	61	−6.8 ± 6.8	−4.4 ± 4.0
LS mean change from baseline [95% CI]		−12.3 [−13.9, −10.7]	−6.4 [−7.3, −5.4]		−9.4 [−11.0, −7.8]	−5.4 [−6.3, −4.5]		−13.1 [−16.0, −10.3]	−7.0 [−8.7, −5.3]		−12.5 [−15.3, −9.7]	−6.7 [−8.3, −5.0]		−11.3 [−13.1, −9.6]	−5.7 [−6.7, −4.7]		−6.6 [−8.4, −4.8]	−4.2 [−5.2, −3.2]
Difference in LS mean change from baseline [95% CI]		−2.9 [−5.2, −0.7]	−1.0 [−2.3, 0.3]		–	–		−0.7 [−4.7, 3.3]	−0.3 [−2.7, 2.0]		–	–		−4.7 [−7.3, −2.2]	−1.5 [−2.9, −0.1]		–	–
EOT	119	127.4 ± 12.5	78.2 ± 8.8	115	130.5 ± 11.2	80.1 ± 8.5	53	127.4 ± 13.9	78.1 ± 8.7	54	128.9 ± 12.1	80.0 ± 9.3	66	127.3 ± 11.4	78.3 ± 8.9	61	131.9 ± 10.3	80.2 ± 7.8

Change from baseline	119	−11.8 ± 10.6	−5.8 ± 5.9	115	−9.7 ± 10.3	−5.7 ± 6.0	53	−13.2 ± 11.5	−6.8 ± 6.3	54	−13.0 ± 12.4	−7.1 ± 7.5	66	−10.6 ± 9.8	−5.0 ± 5.4	61	−6.8 ± 6.8	−4.4 ± 4.0
LS mean change from baseline [95% CI]		−12.1 [−13.7, −10.5]	−6.2 [−7.1, −5.2]		−9.6 [−11.2, −8.0]	−5.5 [−6.4, −4.5]		−13.4 [−16.2, −10.6]	−7.1 [−8.7, −5.5]		−12.8 [−15.6, −10.0	−6.9 [−8.5, −5.2]		−10.7 [−12.5, −8.9]	−5.2 [−6.3, −4.2]		−6.7 [−8.5, −4.8]	−4.2 [−5.3, −3.1]
Difference in LS mean change from baseline [95% CI]		−2.5 [−4.8, −0.2]	−0.7 [−2.0, 0.6]		–	–		−0.6 [−4.5, 3.3]	−0.3 [−2.6, 2.0]		–	–		−4.1 [−6.7, −1.5]	−1.0 [−2.5, 0.5]		–	–
Bedtime home BP																		
Baseline	112	135.0 ± 15.1	79.7 ± 10.0	110	135.2 ± 13.6	80.0 ± 10.9	51	134.5 ± 17.2	79.7 ± 9.6	52	137.3 ± 15.8	81.0 ± 11.1	61	135.4 ± 13.3	79.6 ± 10.4	58	133.3 ± 11.1	79.1 ± 10.7
Week 12	110	124.0 ± 12.9	73.5 ± 9.8	112	125.9 ± 13.4	74.8 ± 9.3	51	123.3 ± 15.4	72.7 ± 9.9	52	123.6 ± 14.8	74.1 ± 10.3	59	124.6 ± 10.4	74.2 ± 9.8	60	127.8 ± 11.8	75.3 ± 8.5
Change from baseline	105	−11.9 ± 10.5 [−13.9, −9.9]***	−6.8 ± 6.3 [−8.0, −5.6]***	108	−9.3 ± 10.7 [−11.3, −7.2]***	−5.0 ± 7.0 [−6.3, −3.6]***	49	−12.6 ± 11.7 [−15.9, −9.2]***	−7.6 ± 7.4 [−9.7, −5.5]***	50	−13.1 ± 12.0 [−16.5, −9.7]***	−6.5 ± 7.8 [−8.7, −4.2]***	56	−11.3 ± 9.5 [−13.8, −8.7]***	−6.1 ± 5.2 [−7.5, −4.7]***	58	−6.0 ± 8.3 [−8.1, −3.8]***	−3.7 ± 6.0 [−5.3, −2.1]***
EOT	117	124.2 ± 13.2	73.6 ± 9.6	114	125.9 ± 13.3	74.8 ± 9.3	53	123.0 ± 15.3	72.7 ± 9.7	54	123.8 ± 14.6	74.2 ± 10.1	64	125.2 ± 11.2	74.3 ± 9.5	60	127.8 ± 11.8	75.3 ± 8.5
Change from baseline	112	−11.4 ± 10.5 [−13.4, −9.4]***	−6.4 ± 6.4 [−7.6, −5.2]***	110	−9.6 ± 10.9 [−11.6, −7.5]***	−5.2 ± 7.1 [−6.5, −3.8]***	51	−12.4 ± 11.5 [−15.7, −9.2]***	−7.5 ± 7.3 [−9.6, −5.5]***	52	−13.6 ± 12.0 [−16.9, −10.2]***	−6.8 ± 7.9 [−9.0, −4.6]***	61	−10.5 ± 9.7 [−13.0, −8.0]***	−5.5 ± 5.4 [−6.9, −4.1]***	58	−6.0 ± 8.3 [−8.1, −3.8]***	−3.7 ± 6.0 [−5.3, −2.1]***

Office BP																		
Baseline	119	141.8 ± 14.9	80.8 ± 11.6	115	141.7 ± 15.8	81.1 ± 10.6	53	144.0 ± 17.3	80.6 ± 11.1	54	145.3 ± 18.1	83.0 ± 9.5	66	140.0 ± 12.5	80.9 ± 12.1	61	138.5 ± 12.9	79.3 ± 11.3
Week 12	113	129.6 ± 15.9	75.5 ± 11.1	112	131.9 ± 15.5	76.3 ± 10.7	51	130.3 ± 18.4	74.7 ± 10.4	52	134.3 ± 16.0	77.3 ± 10.5	62	129.0 ± 13.5	76.1 ± 11.7	60	129.7 ± 14.8	75.3 ± 10.9
Change from baseline	113	−12.3 ± 13.0 [−14.7, −9.9]***	−5.4 ± 8.5 [−7.0, −3.8]***	112	−9.3 ± 12.6 [−11.6, −6.9]***	−4.8 ± 8.8 [−6.5, −3.2]***	51	−14.2 ± 14.0 [−18.1, −10.3]***	−5.7 ± 8.9 [−8.2, −3.2]***	52	−9.7 ± 12.2 [−13.1, −6.3]***	−5.4 ± 9.3 [−8.0, −2.8]***	62	−10.8 ± 11.9 [−13.8, −7.7]***	−5.1 ± 8.2 [−7.1, −3.0]***	60	−9.0 ± 13.0 [−12.3, −5.6]***	−4.3 ± 8.4 [−6.5, −2.1]***
EOT	119	129.5 ± 15.8	75.5 ± 11.0	115	132.1 ± 15.9	76.2 ± 10.9	53	129.6 ± 18.5	74.8 ± 10.2	54	135.3 ± 16.5	77.6 ± 10.6	66	129.5 ± 13.5	76.1 ± 11.7	61	129.3 ± 14.9	75.1 ± 11.1
Change from baseline	119	−12.3 ± 12.9 [−14.6, −10.0]***	−5.3 ± 8.4 [−6.8, −3.8]***	115	−9.5 ± 12.4 [−11.8, −7.2]***	−4.8 ± 8.5 [−6.4, −3.2]***	53	−14.5 ± 13.8 [−18.3, −10.7]***	−5.9 ± 8.7 [−8.3, −3.5]***	54	−10.0 ± 12.1 [−13.3, −6.7]***	−5.5 ± 9.0 [−7.9, −3.0]***	66	−10.5 ± 12.0 [−13.5, −7.6]***	−4.8 ± 8.2 [−6.8, −2.8]***	61	−9.1 ± 12.8 [−12.4, −5.9]***	−4.2 ± 8.1 [−6.3, −2.2]***

Data are mean ± SD unless otherwise specified.

***P <0.001 versus baseline, paired t-test.

LS mean change were calculated for morning home BP at Week 12 and EOT.

LS mean change and 95% CIs were calculated using the analysis of covariance model, with morning home SBP/DBP change from baseline as the objective variable; treatment group as the explanatory variable; and baseline BP, baseline antihypertensive medication, and baseline age as covariates.

Data for the ARB and CCB subgroups are from a post hoc analysis.

ARB angiotensin II receptor blocker, *CCB* calcium channel blocker, *BP* blood pressure, *CI* confidence interval, *DBP* diastolic blood pressure, *EOT* end of treatment, *LS* least squares, *SBP* systolic blood pressure, *SD* standard deviation.

Supplementary Table 3. Change from baseline in BP by baseline UACR (full analysis set)

	UACR <30 mg/gCr						UACR ≥30 mg/gCr					
	Esaxerenone			Trichlormethiazide			Esaxerenone			Trichlormethiazide		
	n	SBP, mmHg	DBP, mmHg	n	SBP, mmHg	DBP, mmHg	n	SBP, mmHg	DBP, mmHg	n	SBP, mmHg	DBP, mmHg
Morning home BP												
Baseline	69	135.5 ± 11.8	83.1 ± 7.7	62	137.9 ± 10.8	85.9 ± 8.0	50	144.1 ± 16.2	85.3 ± 11.1	53	142.9 ± 12.7	85.7 ± 10.4
Week 12	66	124.7 ± 9.9	77.3 ± 8.4	62	127.9 ± 10.1	79.9 ± 7.9	46	130.8 ± 13.6	79.2 ± 9.6	51	133.7 ± 11.8	80.3 ± 9.4
Change from baseline	66	−11.5 ± 11.1	−5.9 ± 5.8	62	−10.0 ± 10.3	−5.9 ± 5.4	46	−12.9 ± 9.5	−6.3 ± 5.5	51	−8.6 ± 9.8	−5.0 ± 6.5
LS mean change from baseline [95% CI]		−12.0 [−14.1, −9.9]	−6.5 [−7.7, −5.2]		−9.4 [−11.6, −7.3]	−5.4 [−6.6, −4.1]		−12.9 [−15.4, −10.5]	−6.2 [−7.7, −4.8]		−9.2 [−11.5, −6.8]	−5.2 [−6.7, −3.8]
Difference in LS mean change from baseline [95% CI]		−2.6 [−5.6, 0.5]	−1.1 [−2.8, 0.7]		–	–		−3.8 [−7.1, −0.4	−1.0 [−3.1, 1.0		–	–
EOT	69	124.4 ± 10.4	77.4 ± 8.4	62	127.9 ± 10.1	79.9 ± 7.9	50	131.4 ± 14.0	79.3 ± 9.3	53	133.5 ± 11.7	80.3 ± 9.2
Change from baseline	69	−11.0 ± 11.3	−5.7 ± 6.0	62	−10.0 ± 10.3	−5.9 ± 5.4	50	−12.8 ± 9.6	−6.0 ± 5.7	53	−9.4 ± 10.4	−5.4 ± 6.7
LS mean change from baseline [95% CI]		−11.7 [−13.8, −9.6]	−6.2 [−7.5, −5.0]		−9.2 [−11.5, −7.0]	−5.3 [−6.6, −4.1]		−13.0 [−15.4, −10.5]	−6.1 [−7.6, −4.6]		−9.9 [−12.3, −7.6]	−5.5 [−7.0, −4.1]
Difference in LS mean change from baseline [95% CI]		−2.5 [−5.5, 0.6]	−0.9 [−2.7, 0.9]		–	–		−3.1 [−6.4, 0.3]	−0.6 [−2.6, 1.5]		–	–
Bedtime home BP												
Baseline	69	132.4 ± 13.3	78.4 ± 9.1	60	132.3 ± 11.0	79.6 ± 10.5	43	139.0 ± 17.0	81.7 ± 11.0	50	138.7 ± 15.6	80.5 ± 11.4
Week 12	66	121.6 ± 10.2	72.1 ± 8.7	61	121.9 ± 11.2	73.7 ± 9.4	44	127.7 ± 15.6	75.6 ± 11.0	51	130.6 ± 14.3	76.0 ± 9.2

Change from baseline	66	−11.5 ± 11.0 [−14.2, −8.8]***	−6.4 ± 6.7 [−8.1, −4.8]***	60	−10.5 ± 10.1 [−13.1, −7.9]***	−5.9 ± 5.9 [−7.4, −4.4]***	39	−12.5 ± 9.8 [−15.7, −9.3]***	−7.4 ± 5.6 [−9.2, −5.6]***	48	−7.8 ± 11.4 [−11.1, −4.5]***	−3.8 ± 8.1 [−6.2, −1.5]**
EOT	69	121.4 ± 10.2	72.2 ± 8.6	61	121.9 ± 11.2	73.7 ± 9.4	48	128.3 ± 15.9	75.6 ± 10.6	53	130.5 ± 14.1	76.0 ± 9.1
Change from baseline	69	−11.1 ± 11.1 [−13.7, −8.4]***	−6.2 ± 6.8 [−7.8, −4.6]***	60	−10.5 ± 10.1 [−13.1, −7.9]***	−5.9 ± 5.9 [−7.4, −4.4]***	43	−11.9 ± 9.6 [−14.9, −8.9]***	−6.8 ± 5.8 [−8.6, −5.1]***	50	−8.5 ± 11.8 [−11.8, −5.2]***	−4.3 ± 8.3 [−6.7, −1.9]***
Office BP												
Baseline	69	140.6 ± 14.0	81.1 ± 11.7	62	136.2 ± 11.9	80.7 ± 10.2	50	143.5 ± 15.9	80.3 ± 11.6	53	148.1 ± 17.5	81.5 ± 11.2
Week 12	67	127.3 ± 12.8	74.5 ± 10.7	62	126.9 ± 12.6	74.9 ± 10.4	46	133.0 ± 19.1	76.9 ± 11.5	50	138.0 ± 16.6	77.9 ± 10.9
Change from baseline	67	−13.3 ± 12.2 [−16.3, −10.3]***	−6.5 ± 7.7 [−8.4, −4.6]***	62	−9.2 ± 11.1 [−12.1, −6.4]***	−5.8 ± 9.1 [−8.1, −3.4]***	46	−11.0 ± 13.9 [−15.1, −6.8]***	−3.7 ± 9.4 [−6.5, −0.9]**	50	−9.4 ± 14.3 [−13.4, −5.3]***	−3.6 ± 8.3 [−6.0, −1.3]**
EOT	69	127.1 ± 12.9	74.6 ± 10.7	62	126.7 ± 12.8	74.9 ± 10.6	50	132.8 ± 18.8	76.8 ± 11.4	53	138.5 ± 16.9	77.8 ± 11.1
Change from baseline	69	−13.4 ± 12.1 [−16.3, −10.5]***	−6.6 ± 7.6 [−8.4, −4.7]***	62	−9.5 ± 11.0 [−12.3, −6.7]***	−5.8 ± 8.9 −8.0, −3.5]***	50	−10.7 ± 13.9 [−14.7, −6.8]***	−3.5 ± 9.2 [−6.1, −0.9]**	53	−9.6 ± 14.0 [−13.5, −5.8]***	−3.7 ± 8.0 [−5.9, −1.5]**

Data are mean ± SD unless otherwise specified.

**P <0.01, ***P <0.001 versus baseline, paired t-test.

LS mean change were calculated for morning home BP at Week 12 and EOT.

LS mean change and 95% CIs were calculated using the analysis of covariance model, with morning home SBP/DBP change from baseline as the objective variable; treatment group as the explanatory variable; and baseline BP, baseline antihypertensive medication, and baseline age as covariates.

Data are from a post hoc analysis.

BP blood pressure, *CI* confidence interval, *DBP* diastolic blood pressure, *EOT* end of treatment, *LS* least squares, *SBP* systolic blood pressure, *SD* standard deviation, *T2DM* type 2 diabetes mellitus, *UACR* urinary albumin-to-creatinine ratio.

Supplementary Table 4. Change from baseline in BP in the population without T2DM (full analysis set)

	Esaxerenone			Trichlormethiazide		
	**n**	**SBP, mmHg**	**DBP, mmHg**	**n**	**SBP, mmHg**	**DBP, mmHg**
Morning home BP						
Baseline	176	140.7 ± 15.4	88.7 ± 9.4	175	139.0 ± 13.9	87.1 ± 9.5
Week 12	171	128.6 ± 13.7	82.0 ± 9.4	172	129.8 ± 12.7	81.6 ± 10.1
Change from baseline	171	−12.0 ± 10.4	−6.6 ± 5.7	172	−9.4 ± 8.5	−5.5 ± 4.9
LS mean change from baseline [95% CI]		−12.2 [−13.5, −11.0]	−6.8 [−7.5, −6.1]		−10.1 [−11.4, −8.9]	−6.0 [−6.7, −5.3]
Difference in LS mean change from baseline [95% CI]		−2.1 [−3.8, −0.4]	−0.8 [−1.8, 0.2]			
EOT	175	128.6 ± 13.7	82.0 ± 9.4	175	129.6 ± 12.8	81.6 ± 10.1
Change from baseline	175	−12.1 ± 10.6	−6.7 ± 5.7	175	−9.4 ± 8.4	−5.5 ± 4.9
LS mean change from baseline [95% CI]		−12.3 [−13.6, −11.1]	−6.8 [−7.5, −6.1]		−10.2 [−11.5, −9.0]	−6.1 [−6.8, −5.3]
Difference in LS mean change from baseline [95% CI]		−2.1 [−3.8, −0.4]	−0.8 [−1.8, 0.2]			
Bedtime home BP						
Baseline	169	134.5 ± 16.3	82.7 ± 10.6	172	133.9 ± 14.3	82.1 ± 10.7

Week 12	170	123.6 ± 13.6	76.7 ± 9.9	171	125.7 ± 13.5	76.7 ± 10.4
Change from baseline	164	−11.0 ± 10.5<br>[−12.6, −9.3]***	−6.1 ± 6.0<br>[−7.0, −5.2]***	169	−8.4 ± 9.7<br>[−9.9, −6.9]***	−5.4 ± 6.0<br>[−6.3, −4.5]***
EOT	174	123.5 ± 13.6	76.6 ± 9.9	174	125.4 ± 13.6	76.7 ± 10.3
Change from baseline	168	−10.9 ± 10.6<br>[−12.5, −9.3]***	−6.1 ± 6.0<br>[−7.0, −5.1]***	172	−8.5 ± 9.7<br>[−9.9, −7.0]***	−5.4 ± 5.9<br>[−6.3, −4.5]***
Office BP						
Baseline	176	145.1 ± 17.4	85.3 ± 11.3	175	143.4 ± 14.7	85.0 ± 12.9
Week 12	170	131.4 ± 16.0	78.1 ± 11.7	172	133.8 ± 14.0	80.0 ± 12.6
Change from baseline	170	−13.6 ± 13.3<br>[−15.6, −11.6]***	−7.0 ± 9.0<br>[−8.4, −5.7]***	172	−9.7 ± 13.0<br>[−11.7, −7.7]***	−5.0 ± 8.7<br>[−6.3, −3.7]***
EOT	175	131.5 ± 15.7	78.0 ± 11.6	175	133.8 ± 14.1	80.0 ± 12.7
Change from baseline	175	−13.6 ± 13.3<br>[−15.5, −11.6]***	−7.1 ± 9.0<br>[−8.5, −5.8]***	175	−9.7 ± 13.0<br>[−11.6, −7.7]***	−5.0 ± 8.7<br>[−6.3, −3.7]***

Data are mean ± SD unless otherwise specified.

***P <0.001 versus baseline, paired *t*-test.

LS mean change were calculated for morning home BP at Week 12 and EOT.

LS mean change and 95% CIs were calculated using the analysis of covariance model, with morning home SBP/DBP change from baseline as the objective variable; treatment group as the explanatory variable; and baseline BP, baseline antihypertensive medication, and baseline age as covariates.

BP blood pressure, *CI* confidence interval, *DBP* diastolic blood pressure, *EOT* end of treatment, *LS* least squares, *SBP* systolic blood pressure, *SD* standard deviation, *T2DM* type 2 diabetes mellitus.

Supplementary Table 5. Change in UACR from baseline to Week 12 in the overall population and in subgroups by baseline antihypertensive agent (full analysis set)

	Overall				ARB				CCB			
	Esaxerenone		Trichlormethiazide		Esaxerenone		Trichlormethiazide		Esaxerenone		Trichlormethiazide	
**UACR, mg/gCr**	**n**		**n**		**n**		**n**		**n**		**n**	
Baseline	119	165.96 ± 442.12	115	156.56 ± 637.29	53	192.41 ± 540.65	54	230.78 ± 916.54	66	144.72 ± 346.38	61	90.86 ± 142.36
Week 4	119	98.43 ± 238.05	115	118.61 ± 500.46	53	117.69 ± 323.90	54	189.84 ± 721.38	66	82.95 ± 135.66	61	55.54 ± 87.11
Change from baseline	119	−67.53 ± 267.66	115	−37.95 ± 243.82	53	−74.71 ± 248.71	54	−40.93 ± 346.89	66	−61.77 ± 283.72	61	−35.31 ± 81.53
Percentage change in geometric mean from baseline [95% CI]		−27.7 [−36.9, −17.3]***		−30.2 [−38.9, −20.3]***		−32.0 [−45.0, −15.9]***		−28.6 [−42.1, −11.8]**		−24.1 [−36.6, −9.2]**		−31.7 [−42.5, −18.7]***
Week 8	115	90.72 ± 234.28	114	88.04 ± 374.87	52	104.64 ± 298.66	53	137.86 ± 542.56	63	79.23 ± 165.09	61	44.75 ± 73.75
Change from baseline	115	−53.38 ± 208.61	114	−69.12 ± 276.91	52	−91.00 ± 293.54	53	−95.62 ± 395.26	63	−22.33 ± 83.51	61	−46.10 ± 88.64
Percentage change in geometric mean from baseline [95% CI]		−32.7 [−41.6, −22.5]***		−40.0 [−47.2, −31.8]***		−29.9 [−43.6, −12.9]**		−36.8 [−47.0, −24.6]***		−35.0 [−46.3, −21.2]***		−42.7 [−52.5, −30.8]***

Week 12	113	102.15 ± 357.67	112	91.89 ± 399.08	51	171.65 ± 517.89	52	152.20 ± 578.89	62	44.98 ± 87.65	60	39.61 ± 63.10
Change from baseline	113	−34.04 ± 254.17	112	−67.34 ± 298.40	51	−26.02 ± 365.46	52	−84.88 ± 425.28	62	−40.64 ± 95.08	60	−52.14 ± 103.34
Percentage change in geometric mean from baseline [95% CI]		−35.9 [−46.3, −23.5]***		−41.4 [−49.6, −32.0]***		−24.7 [−44.3, 1.9]		−31.4 [−44.5, −15.3]***		−43.9 [−54.3, −31.1]***		−49.0 [−58.6, −37.1]***

Data are geometric mean ± SD unless otherwise specified.

**P* <0.05, ***P* <0.01, ****P* <0.001 versus baseline, paired *t*-test.

Data for the ARB and CCB subgroups are from a post hoc analysis.

ARB angiotensin II receptor blocker, *CCB* calcium channel blocker, *CI* confidence interval, *SD* standard deviation, *UACR* urinary albumin-to-creatinine ratio.

Supplementary Table 6. Change in UACR from baseline to Week 12 by baseline UACR (full analysis set)

	UACR <30 mg/gCr				UACR ≥30 mg/gCr			
	Esaxerenone		Trichlormethiazide		Esaxerenone		Trichlormethiazide	
**UACR, mg/gCr**	**n**		**n**		**n**		**n**	
Baseline	69	11.31 ± 6.43	62	13.74 ± 7.47	50	379.37 ± 624.91	53	323.63 ± 915.17
Week 4	69	10.10 ± 8.82	62	14.25 ± 11.51	50	220.32 ± 331.98	53	240.68 ± 721.63
Change from baseline	69	−1.22 ± 8.40	62	0.51 ± 8.99	50	−159.05 ± 397.10	53	−82.95 ± 355.54
Percentage change in geometric mean from baseline [95% CI]		−18.2 [−28.9, −5.9]**		−8.2 [−20.1, 5.6]		−39.1 [−52.9, −21.3]***		−49.4 [−59.0, −37.5]***
Week 8	67	9.90 ± 8.67	62	11.45 ± 8.35	48	203.52 ± 332.74	52	179.36 ± 543.73
Change from baseline	67	−1.10 ± 7.51	62	−2.29 ± 7.26	48	−126.36 ± 310.07	52	−148.81 ± 397.41
Percentage change in geometric mean from baseline [95% CI]		−18.5 [−29.5, −5.7]**		−22.3 [−32.7, −10.4]***		−48.6 [−60.4, −33.3]***		−55.9 [−63.7, −46.4]***
Week 12	67	9.87 ± 11.04	62	12.10 ± 13.15	46	236.55 ± 535.80	50	190.81 ± 585.26
Change from baseline	67	−1.13 ± 10.95	62	−1.64 ± 12.72	46	−81.98 ± 395.79	50	−148.82 ± 435.05

Percentage change in geometric mean from baseline [95% CI]	−20.3 [−33.4, −4.7]*	−22.7 [−33.7, −9.8]**	−53.3 [−66.6, −34.8]***	−58.5 [−67.6, −46.9]***

Data are geometric mean ± SD unless otherwise specified.

*P <0.05, **P <0.01, ***P <0.001 versus baseline, paired *t*-test.

Data are from a post hoc analysis.

CI confidence interval, *T2DM* type 2 diabetes mellitus, *SD* standard deviation, *UACR* urinary albumin-to-creatinine ratio.

Supplementary Table 7. Change in UACR from baseline to Week 12 in the population without T2DM (full analysis set)

	Esaxerenone		Trichlormethiazide	
**UACR, mg/gCr**	**n**		**n**	
Baseline	176	82.67 ± 506.16	175	65.08 ± 172.48
Week 4	176	63.95 ± 494.60	175	38.33 ± 103.85
Change from baseline	176	−18.72 ± 73.15	175	−26.74 ± 146.51
Percentage change in geometric mean from baseline [95% CI]		−36.4 [−42.9, −29.1]***		−28.1 [−36.0, −19.2]***
Week 8	172	22.87 ± 61.23	172	33.94 ± 100.77
Change from baseline	172	−22.65 ± 83.56	172	−32.13 ± 162.46
Percentage change in geometric mean from baseline [95% CI]		−41.8 [−48.6, −34.1]***		−32.7 [−40.2, −24.2]***
Week 12	170	19.98 ± 35.25	172	29.93 ± 82.02
Change from baseline	170	−25.93 ± 96.43	172	−36.14 ± 152.63
Percentage change in geometric mean from baseline [95% CI]		−40.9 [−48.3, −32.3]***		−42.2 [−48.5, −35.1]***

Data are geometric mean ± SD unless otherwise specified.

***P <0.001 versus baseline, paired t-test.

CI confidence interval, *T2DM* type 2 diabetes mellitus, *SD* standard deviation, *UACR* urinary albumin-to-creatinine ratio.

Supplementary Table 8. TEAEs in the overall population and in subgroups by baseline antihypertensive agent (safety analysis set)

	Overall		ARB		CCB	
	**Esaxerenone n = 122**	**Trichlormethiazide n = 120**	**Esaxerenone n = 55**	**Trichlormethiazide n = 55**	**Esaxerenone n = 67**	**Trichlormethiazide n = 65**
Any TEAEs	40 (32.8)	33 (27.5)	18 (32.7)	18 (32.7)	22 (32.8)	15 (23.1)
TEAEs occurring in ≥2 patients in either group[1]						
Nasopharyngitis	2 (1.6)	4 (3.3)	1 (1.8)	1 (1.8)	1 (1.5)	3 (4.6)
Hyperuricemia	1 (0.8)	4 (3.3)	0	3 (5.5)	1 (1.5)	1 (1.5)
Blood potassium increased	4 (3.3)	0	2 (3.6)	0	2 (3.0)	0
eGFR decreased	1 (0.8)	3 (2.5)	0	2 (3.6)	1 (1.5)	1 (1.5)
Abdominal pain upper	0	3 (2.5)	0	3 (5.5)	0	0
Arthralgia	1 (0.8)	2 (1.7)	1 (1.8)	2 (3.6)	0	0
Back pain	2 (1.6)	1 (0.8)	0	0	2 (3.0)	1 (1.5)
Blood UA increased	0	3 (2.5)	0	2 (3.6)	0	1 (1.5)
Bronchitis	2 (1.6)	1 (0.8)	0	0	2 (3.0)	1 (1.5)
COVID-19	1 (0.8)	2 (1.7)	1 (1.8)	1 (1.8)	0	1 (1.5)
Dizziness	2 (1.6)	1 (0.8)	1 (1.8)	0	1 (1.5)	1 (1.5)
Pharyngitis	2 (1.6)	1 (0.8)	1 (1.8)	1 (1.8)	1 (1.5)	0
UACR increased	2 (1.6)	1 (0.8)	1 (1.8)	1 (1.8)	1 (1.5)	0
Blood sodium decreased	0	2 (1.7)	0	1 (1.8)	0	1 (1.5)

Headache	2 (1.6)	0	0	0	2 (3.0)	0
T2DM	0	2 (1.7)	0	0	0	2 (3.1)
Blood potassium decreased	0	1 (0.8)	0	0	0	1 (1.5)
Blood sodium increased	0	0	0	0	0	0

Data are n (%). TEAEs are coded using MedDRA/J version 25.1.

Data for the ARB and CCB subgroups are from a post hoc analysis.

[1]Except for blood potassium decreased and blood sodium increased.

ARB angiotensin II receptor blocker, *CCB* calcium channel blocker, *eGFR* estimated glomerular filtration rate, *MedDRA/J* Medical Dictionary for Regulatory Activities for Japanese, *TEAE* treatment-emergent adverse event, *T2DM* type 2 diabetes mellitus, *UA* uric acid, *UACR* urinary albumin-to-creatinine ratio.

Supplementary Table 9. TEAEs by baseline UACR (safety analysis set)

	UACR <30 mg/gCr		UACR ≥30 mg/gCr	
	**Esaxerenone**<br>**n = 71**	**Trichlormethiazide**<br>**n = 64**	**Esaxerenone**<br>**n = 51**	**Trichlormethiazide**<br>**n = 56**
Any TEAEs	21 (29.6)	13 (20.3)	19 (37.3)	20 (35.7)
TEAEs occurring in ≥2 patients in either group[1]				
Nasopharyngitis	1 (1.4)	2 (3.1)	1 (2.0)	2 (3.6)
Hyperuricemia	1 (1.4)	2 (3.1)	0	2 (3.6)
Blood potassium increased	3 (4.2)	0	1 (2.0)	0
eGFR decreased	0	0	1 (2.0)	3 (5.4)
Abdominal pain upper	0	1 (1.6)	0	2 (3.6)
Arthralgia	1 (1.4)	1 (1.6)	0	1 (1.8)
Back pain	2 (2.8)	0	0	1 (1.8)
Blood UA increased	0	1 (1.6)	0	2 (3.6)
Bronchitis	0	0	2 (3.9)	1 (1.8)
COVID-19	1 (1.4)	2 (3.1)	0	0

Dizziness	2 (2.8)	1 (1.6)	0	0
Pharyngitis	2 (2.8)	0	0	1 (1.8)
UACR increased	0	0	2 (3.9)	1 (1.8)
Blood sodium decreased	0	0	0	2 (3.6)
Headache	1 (1.4)	0	1 (2.0)	0
T2DM	0	0	0	2 (3.6)
Blood potassium decreased	0	0	0	1 (1.8)
Blood sodium increased	0	0	0	0

Data are n (%). TEAEs are coded using MedDRA/J version 25.1.

Data are from a post hoc analysis.

[1]Except for arthralgia, headache, blood potassium decreased, and blood sodium increased.

eGFR estimated glomerular filtration rate, *MedDRA/J* Medical Dictionary for Regulatory Activities for Japanese, *TEAE* treatment-emergent adverse event, *T2DM* type 2 diabetes mellitus, *UA* uric acid, *UACR* urinary albumin-to-creatinine ratio.

Supplementary Table 10. Change in serum potassium and $eGFR_{creat}$ from baseline to Week 12 in the overall population and in subgroups by baseline antihypertensive agent (safety analysis set)

	Overall				ARB				CCB			
	Esaxerenone		Trichlormethiazide		Esaxerenone		Trichlormethiazide		Esaxerenone		Trichlormethiazide	
	n	Mean ± SD	n	Mean ± SD	n	Mean ± SD	n	Mean ± SD	n	Mean ± SD	n	Mean ± SD
**Serum potassium (mEq/L)**												
Baseline	118	4.26 ± 0.33	115	4.26 ± 0.35	53	4.35 ± 0.32	52	4.33 ± 0.33	65	4.19 ± 0.33	63	4.20 ± 0.37
Week 2	116	4.40 ± 0.32	113	4.17 ± 0.39	53	4.43 ± 0.32	52	4.24 ± 0.31	63	4.37 ± 0.32	61	4.11 ± 0.44
Change from baseline	115	0.14 ± 0.31	113	−0.09 ± 0.34	52	0.07 ± 0.29	52	−0.09 ± 0.36	63	0.19 ± 0.33	61	−0.10 ± 0.33
Week 4	116	4.31 ± 0.39	110	4.16 ± 0.35	52	4.29 ± 0.37	50	4.22 ± 0.34	64	4.33 ± 0.40	60	4.10 ± 0.34
Change from baseline	115	0.05 ± 0.37	110	−0.10 ± 0.33	51	−0.05 ± 0.31	50	−0.09 ± 0.35	64	0.13 ± 0.39	60	−0.10 ± 0.32
Week 8	112	4.35 ± 0.37	110	4.13 ± 0.35	51	4.37 ± 0.37	50	4.20 ± 0.34	61	4.34 ± 0.37	60	4.07 ± 0.34
Change from baseline	111	0.10 ± 0.37	110	−0.12 ± 0.37	50	0.03 ± 0.39	50	−0.12 ± 0.40	61	0.15 ± 0.35	60	−0.13 ± 0.34
Week 12	110	4.31 ± 0.36	107	4.05 ± 0.38	50	4.30 ± 0.41	49	4.19 ± 0.36	60	4.31 ± 0.31	58	3.94 ± 0.35
Change from baseline	109	0.05 ± 0.36	107	−0.21 ± 0.38	49	−0.04 ± 0.37	49	−0.13 ± 0.42	60	0.13 ± 0.33	58	−0.27 ± 0.33
**$eGFR_{creat}$ (mL/min/1.73 m^2)**												
Baseline	122	72.75 ± 17.43	118	70.34 ± 17.53	55	74.39 ± 17.98	55	67.00 ± 19.48	67	71.39 ± 16.98	63	73.27 ± 15.20
Week 2	120	68.44 ± 15.87	118	68.20 ± 17.13	54	69.52 ± 15.35	55	66.10 ± 19.35	66	67.56 ± 16.35	63	70.03 ± 14.83
Change from baseline	120	−4.35 ± 7.97	116	−2.36 ± 8.45	54	−5.27 ± 10.10	55	−0.89 ± 8.92	66	−3.59 ± 5.64	61	−3.67 ± 7.84
Week 4	119	68.28 ± 16.56	115	67.81 ± 16.47	53	69.21 ± 17.13	53	65.37 ± 17.74	66	67.53 ± 16.19	62	69.90 ± 15.14
Change from baseline	119	−4.53 ± 8.83	113	−2.94 ± 8.44	53	−5.66 ± 10.87	53	−1.76 ± 8.60	66	−3.63 ± 6.72	60	−3.99 ± 8.22
Week 8	115	66.96 ± 16.98	115	67.80 ± 18.27	52	69.28 ± 18.35	53	64.89 ± 19.35	63	65.05 ± 15.65	62	70.29 ± 17.07

Change from baseline	115	−5.68 ± 8.88	113	−2.98 ± 9.53	52	−5.73 ± 10.87	53	−2.51 ± 7.46	63	−5.64 ± 6.92	60	−3.39 ± 11.09
Week 12	113	66.21 ± 16.29	112	68.01 ± 16.55	51	67.76 ± 16.68	52	64.99 ± 17.93	62	64.94 ± 15.98	60	70.62 ± 14.91
Change from baseline	113	−6.48 ± 9.11	110	−2.88 ± 9.42	51	−6.88 ± 9.94	52	−3.00 ± 9.25	62	−6.16 ± 8.42	58	−2.77 ± 9.65

P values were not calculated.

Data for the ARB and CCB subgroups are from a post hoc analysis.

ARB angiotensin II receptor blocker, *CCB* calcium channel blocker, $eGFR_{creat}$ creatinine-based estimated glomerular filtration rate, *SD* standard deviation.

Supplementary Table 11. Change in serum potassium and $eGFR_{creat}$ from baseline to Week 12 by baseline UACR (safety analysis set)

	**UACR <30 mg/gCr**				**UACR ≥30 mg/gCr**			
	**Esaxerenone**		**Trichlormethiazide**		**Esaxerenone**		**Trichlormethiazide**	
	n	Mean ± SD	n	Mean ± SD	n	Mean ± SD	n	Mean ± SD
**Serum potassium (mEq/L)**								
Baseline	70	4.28 ± 0.33	61	4.32 ± 0.34	48	4.24 ± 0.34	54	4.19 ± 0.36
Week 2	68	4.44 ± 0.30	60	4.25 ± 0.39	48	4.34 ± 0.34	53	4.08 ± 0.37
Change from baseline	68	0.17 ± 0.33	60	−0.06 ± 0.36	47	0.09 ± 0.29	53	−0.13 ± 0.32
Week 4	68	4.36 ± 0.38	59	4.17 ± 0.34	48	4.24 ± 0.39	51	4.14 ± 0.36
Change from baseline	68	0.09 ± 0.39	59	−0.13 ± 0.37	47	0.00 ± 0.32	51	−0.05 ± 0.29
Week 8	66	4.32 ± 0.35	59	4.16 ± 0.36	46	4.40 ± 0.39	51	4.10 ± 0.33
Change from baseline	66	0.06 ± 0.38	59	−0.15 ± 0.36	45	0.15 ± 0.35	51	−0.09 ± 0.38
Week 12	66	4.27 ± 0.32	59	4.08 ± 0.33	44	4.37 ± 0.39	48	4.01 ± 0.42
Change from baseline	66	0.01 ± 0.35	59	−0.22 ± 0.37	43	0.12 ± 0.36	48	−0.19 ± 0.39
**$eGFR_{creat}$ (mL/min/1.73 m^2)**								
Baseline	71	74.05 ± 17.68	64	73.46 ± 16.91	51	70.94 ± 17.08	54	66.66 ± 17.70
Week 2	70	68.98 ± 15.21	63	70.13 ± 16.38	50	67.69 ± 16.89	55	65.99 ± 17.83
Change from baseline	70	−5.36 ± 8.94	63	−3.49 ± 8.79	50	−2.93 ± 6.18	53	−1.01 ± 7.89

Week 4	69	69.09 ± 16.31	62	70.56 ± 15.62	50	67.16 ± 17.01	53	64.60 ± 17.00
Change from baseline	69	−5.31 ± 10.31	62	−3.32 ± 8.21	50	−3.45 ± 6.18	51	−2.48 ± 8.77
Week 8	67	68.31 ± 17.45	62	69.64 ± 17.22	48	65.09 ± 16.29	53	65.66 ± 19.38
Change from baseline	67	−6.01 ± 9.93	62	−4.24 ± 7.62	48	−5.22 ± 7.25	51	−1.44 ± 11.32
Week 12	67	67.56 ± 15.45	62	72.02 ± 15.26	46	64.25 ± 17.42	50	63.03 ± 16.87
Change from baseline	67	−6.76 ± 9.72	62	−1.87 ± 10.00	46	−6.09 ± 8.22	48	−4.18 ± 8.54

P values were not calculated.

Data are from a post hoc analysis.

$eGFR_{creat}$ creatinine-based estimated glomerular filtration rate, *SD* standard deviation,

T2DM type 2 diabetes mellitus, *UACR* urinary albumin-to-creatinine ratio.

Supplementary Figures

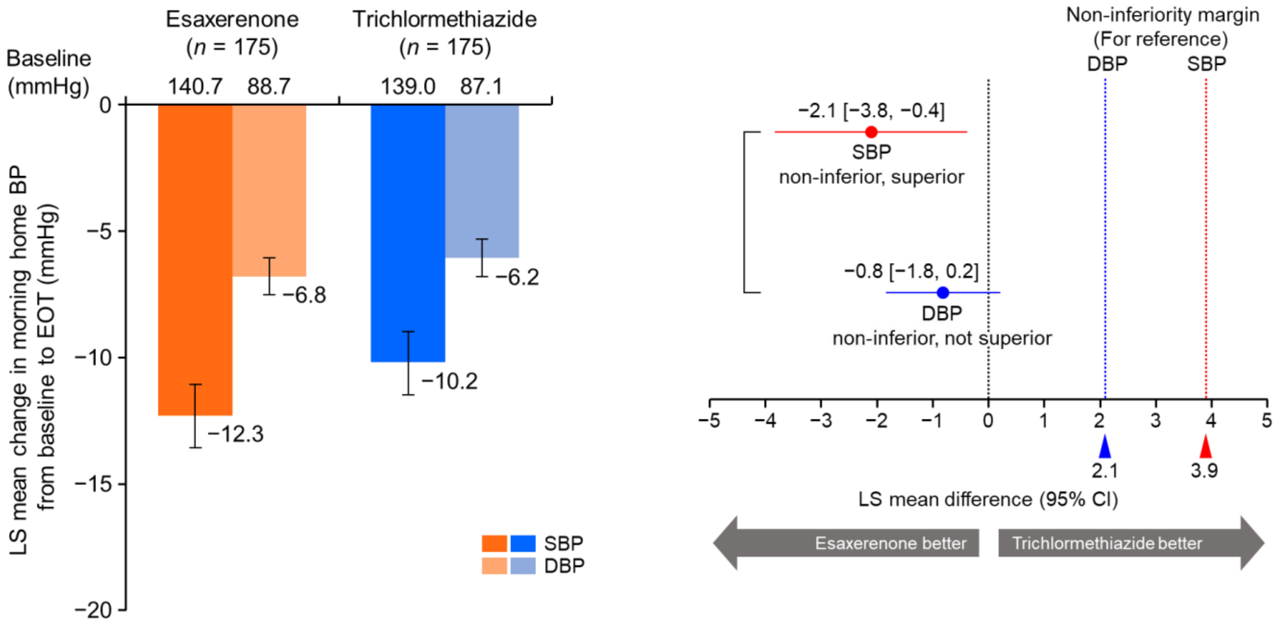

Supplementary Figure 1. Changes from baseline to EOT in morning home BP in the population without T2DM.

The red dotted line (3.9 mmHg) and blue dotted line (2.1 mmHg) indicate the non-inferiority criteria. Data are LS mean (95% CI).

BP blood pressure, *CI* confidence interval, *DBP* diastolic blood pressure, *EOT* end of treatment, *LS* least squares, *SBP* systolic blood pressure, *T2DM* type 2 diabetes mellitus.

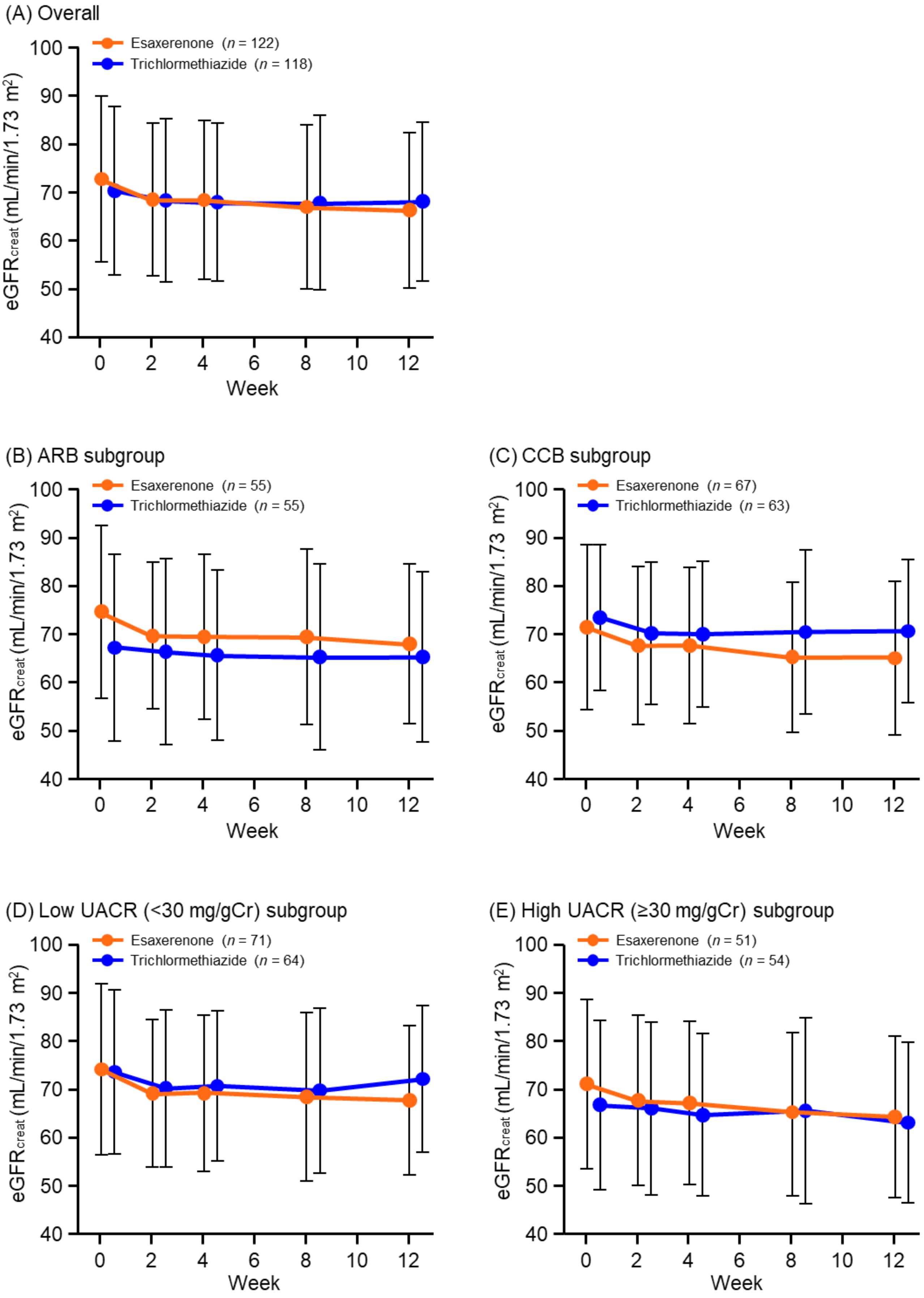

(A) Overall
Esaxerenone (n = 122)
Trichlormethiazide (n = 118)
(B) ARB subgroup
Esaxerenone (n = 55)
Trichlormethiazide (n = 55)
(C) CCB subgroup
Esaxerenone (n = 67)
Trichlormethiazide (n = 63)
(D) Low UACR (<30 mg/gCr) subgroup
Esaxerenone (n = 71)
Trichlormethiazide (n = 64)
(E) High UACR (≥30 mg/gCr) subgroup
Esaxerenone (n = 51)
Trichlormethiazide (n = 54)
eGFRcreat (mL/min/1.73 m2)
100
90
80
70
60
50
40
0
2
4
6
8
10
12
Week

Supplementary Figure 2. Time course changes in $eGFR_{creat}$ (safety analysis set).

(**A**) overall population, (**B**) ARB subgroup, (**C**) CCB subgroup, (**D**) low UACR (<30 mg/gCr) subgroup, and (**E**) high UACR (≥30 mg/gCr) subgroup.

Data are mean ± SD.

Data for the ARB, CCB, and UACR subgroups are from a post hoc analysis.

ARB angiotensin II receptor blocker, *CCB* calcium channel blocker, *$eGFR_{creat}$* creatinine-based estimated glomerular filtration rate, *SD* standard deviation, *UACR* urinary albumin-to-creatinine ratio.

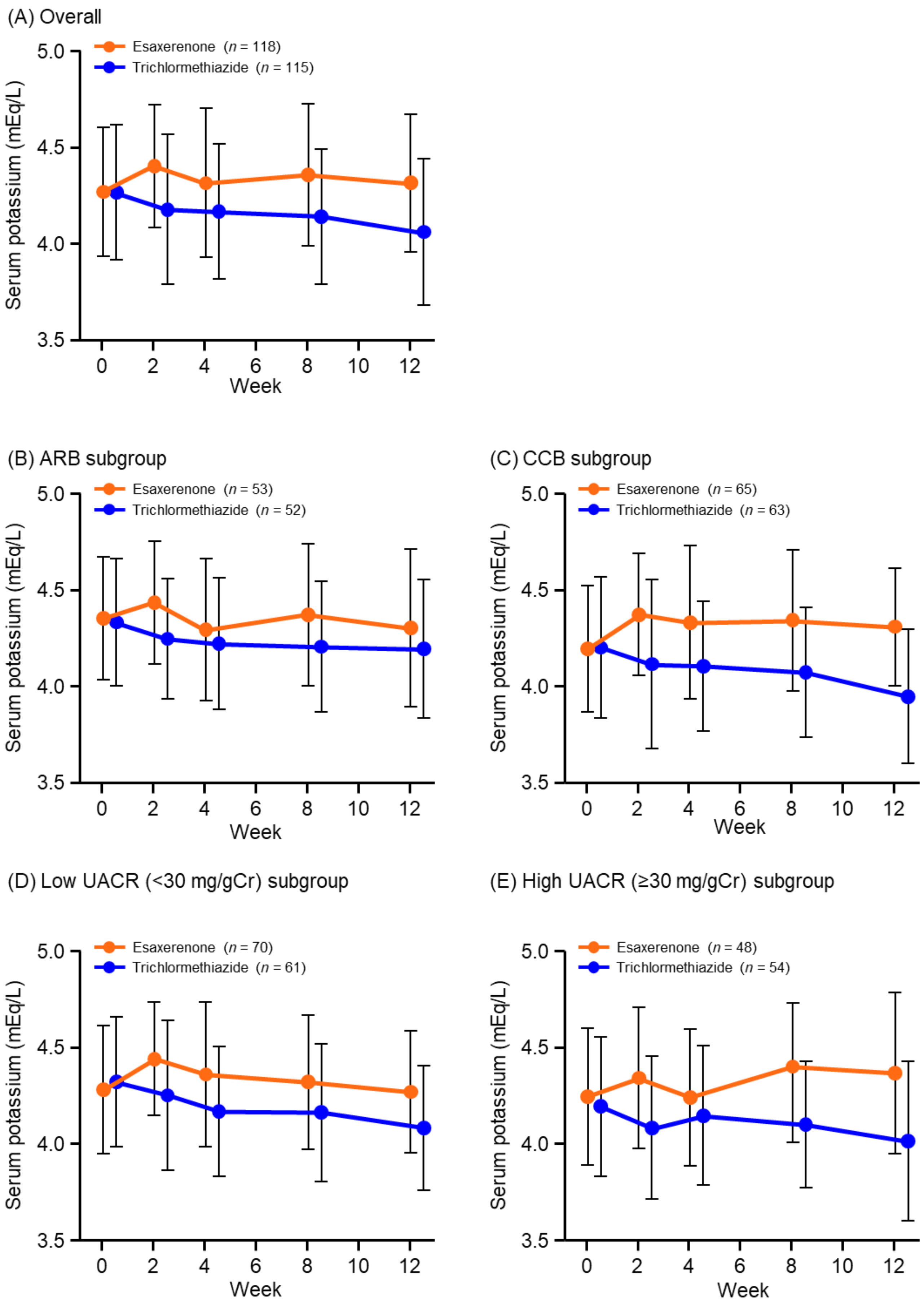

(A) Overall
Esaxerenone (n = 118)
Trichlormethiazide (n = 115)
Serum potassium (mEq/L)
5.0
4.5
4.0
3.5
0
2
4
6
8
10
12
Week
(B) ARB subgroup
Esaxerenone (n = 53)
Trichlormethiazide (n = 52)
Serum potassium (mEq/L)
Week
(C) CCB subgroup
Esaxerenone (n = 65)
Trichlormethiazide (n = 63)
Serum potassium (mEq/L)
Week
(D) Low UACR (<30 mg/gCr) subgroup
Esaxerenone (n = 70)
Trichlormethiazide (n = 61)
Serum potassium (mEq/L)
Week
(E) High UACR (≥30 mg/gCr) subgroup
Esaxerenone (n = 48)
Trichlormethiazide (n = 54)
Serum potassium (mEq/L)
Week

Supplementary Figure 3. Time course changes in serum potassium levels (safety analysis set).

(**A**) overall population, (**B**) ARB subgroup, (**C**) CCB subgroup, (**D**) low UACR (<30 mg/gCr) subgroup, and (**E**) high UACR (≥30 mg/gCr) subgroup.

Data are mean ± SD.

Data for the ARB, CCB, and UACR subgroups are from a post hoc analysis.

ARB angiotensin II receptor blocker, *CCB* calcium channel blocker, *SD* standard deviation, *UACR* urinary albumin-to-creatinine ratio.
